# The hypoxic microenvironment of *Candida albicans* biofilms shapes neutrophil responses

**DOI:** 10.3389/fimmu.2025.1547559

**Published:** 2025-04-22

**Authors:** Magdalena Juszczak, Aleksandra Brankiewicz, Marcin Zawrotniak, Maria Rapala-Kozik

**Affiliations:** ^1^ Department of Comparative Biochemistry and Bioanalytics, Faculty of Biochemistry, Biophysics and Biotechnology, Jagiellonian University, Krakow, Poland; ^2^ Doctoral School of Exact and Natural Sciences, Jagiellonian University, Krakow, Poland

**Keywords:** neutrophils, *Candida albicans*, biofilms, hypoxia, HIF-1α

## Abstract

**Introduction:**

The microenvironment of *Candida albicans* biofilms create a hypoxic microenvironment, which exerts a profound influence on host immune responses during infection. Neutrophils are key defenders against *C. albicans*; however, the impact of biofilm-induced hypoxia on neutrophil function remains unclear.

**Methods:**

We co-cultured human neutrophils in vitro with *C. albicans* biofilms at various stages of maturation, using both wild-type strains and extracellular matrix (ECM)-deficient mutants. Intracellular hypoxia was assessed using a fluorescent oxygen-sensitive probe. Neutrophil effector functions were evaluated by measuring caspase-3/7 activity, stabilization of hypoxia-inducible factor 1-alpha (HIF-1α), and accumulation of the anti-apoptotic Mcl-1 protein. Analyses included also quantification of reactive oxygen species (ROS) production, neutrophil extracellular trap (NET) formation, chemokine secretion (IL-8 and MIP-1β), and neutrophil elastase release. To assess the role of hypoxia signaling in neutrophil responses, cells were treated with the selective HIF-1α inhibitors LW6 and PX478.

**Results:**

Neutrophils infiltrating *C. albicans* wild-type biofilms experience progressive hypoxia, which intensifies with biofilm maturation. This hypoxia results from high fungal metabolic activity and extracellular matrix (ECM) production. Within the biofilm microenvironment, neutrophils exhibit increased stabilization of HIF-1α and Mcl-1, elevated secretion of MIP-1β, IL-8, and reduced caspase 3/7 activity, collectively suggesting a biofilm-induced pro-survival phenotype. Simultaneously, mature biofilms markedly suppress NET formation and ROS production while enhancing degranulation. Comparative analyses using mannan-deficient *C. albicans* mutants highlight the critical role of ECM composition in modulating hypoxia-driven immune responses. Pharmacological inhibition of HIF-1α with LW6 and PX478 partially restores NETosis and ROS production, underscoring the pivotal role of this protein in regulation of neutrophil function.

**Discussion:**

These findings provide novel insights into the impact of biofilm-induced hypoxia on neutrophil responses, identifying HIF-1α as a key regulator of immune adaptation in fungal biofilms. Targeting hypoxia pathways may offer new therapeutic strategies to modulate neutrophil responses and enhance host defenses against fungal infections.

## 1 Introduction

The formation of biofilms by pathogenic microorganisms poses a significant challenge in clinical settings, primarily due to their structural complexity and heightened resistance to conventional antimicrobial therapies. Within biofilms, microorganisms undergo phenotypic shifts characterized by altered growth patterns and gene expression, providing them with specific adaptive advantages over their planktonic form (1–3). These adaptations confer unique physiological and biochemical properties that enhance the biofilm’s resilience and persistence across diverse environments. The formation of biofilms is one of the main virulence mechanisms used by *Candida albicans.* This opportunistic yeast is a frequent cause of fungal infections, ranging from superficial mucosal conditions to severe, life-threatening systemic diseases, particularly in immunocompromised individuals (3, 4).

The development of a fungal infection triggers the recruitment of various immune cells, particularly neutrophils, which serve as the host’s first line of defense (5). Neutrophils employ multiple intracellular and extracellular mechanisms to combat pathogens, mainly phagocytosis, degranulation, reactive oxygen species generation (ROS) and NETosis (6). NETosis is a specialized form of cell death characterized by the release of neutrophil extracellular traps (NETs), which consist of decondensed chromatin and proteins from neutrophil granules (7, 8). Activation of the NETs release is typically dependent on ROS production by the membrane-bound multiprotein NADPH oxidase complex (9). However, some studies suggest that NETosis can also be initiated through ROS-independent pathways in response to certain factors (10, 11). A range of fungal, bacterial, and chemical agents have been implicated in triggering NETosis. In the case of *C. albicans*, β-glucans, aspartyl proteases, quorum-sensing molecules, toxins, and nucleic acids contribute to this activation process (12–15).

Despite their broad spectrum of antimicrobial activity, the mechanisms used by neutrophils may become ineffective or impaired when in contact with biofilm (16, 17). One of the significant factor contributing to impaired neutrophil responses in the presence of biofilms is the physical barrier created by the extracellular matrix (ECM) of the biofilm. The ECM not only restricts neutrophil access to embedded pathogens but also diminishes the efficacy of phagocytosis (18). Moreover, high concentrations of polysaccharides, such as β-glucans within the ECM, have been shown to inhibit NETosis (17, 19). Beyond its physical shielding function, the architecture of biofilms creates unique microenvironments that further challenge immune responses. It has been demonstrated that fungal biofilm can generate localized hypoxic niches, primarily due to the limited diffusion of oxygen through the dense matrix and the high metabolic activity of resident microorganisms (20, 21). *Candida* species adapt to hypoxia through a range of metabolic and genetic mechanisms that allow them to thrive in low-oxygen environments. These adaptations include upregulation of genes associated with anaerobic metabolism, such as those involved in glycolysis and fermentation. *Candida* also modifies its cell membrane composition and increases the expression of specific transcription factors, such as Efg1 (22). The hypoxic conditions facilitate the formation of multispecies biofilm with anaerobic bacteria, enhancing both the biofilm’s pathogenicity and resistance to treatment (23, 24).

Hypoxic environments significantly affect the function of immune cells. In response to low oxygen levels, human cells activate hypoxia-inducible factor 1-alpha (HIF-1α) (25). Under normoxic conditions, HIF-1α is hydroxylated by prolyl hydroxylase (PHD) and subsequently targeted for proteasomal degradation. However, in hypoxic environments, reduced hydroxylase activity leads to HIF-1α stabilization and nuclear translocation, where it regulates the expression of genes involved in cellular adaptation to low oxygen levels (26). In neutrophils, HIF-1α activation orchestrates multiple adaptive responses, including enhanced glycolytic metabolism, modulation of apoptotic pathways, and regulation of inflammatory gene expression (27). Despite these insights, the precise role of hypoxia in modulating neutrophil responses during interactions with *C. albicans* biofilms remains incompletely understood. Therefore, in this study, we aimed to elucidate the impact of hypoxia on neutrophil responses within biofilm environments and to explore the potential for enhancing neutrophil fungicidal activity by targeting this HIF-1α.

## 2 Materials and methods

### 2.1 Yeast strains and culture

*Candida albicans* ATCC 10231 and SC5314 strains were purchased from American Type Culture Collection (ATCC, Manassas, VA, USA). The deletion mutants (*Δbgl2*, *Δmnn9*) were obtained from the Fungal Genetic Stock Center (FGSC), originally provided by Prof. Aron Mitchell’s lab. The *Δefg1/Δcph1* mutant strain was kindly provided by Angela Nobbs. Yeast cultures were maintained in YPD medium (1% yeast extract, 2% soybean peptone, and 2% glucose) (Sigma-Aldrich, St. Louis, MO, USA) at 30°C with continuous shaking (180 rpm) in a MaxQ 6000 orbital rotary shaker (Thermo Fisher Scientific, Waltham, MA, USA). After 16 hours of incubation, cultures reached the stationary phase, and cell density was measured by optical density (OD) at 600 nm.

### 2.2 Biofilm formation

Biofilms (strains ATCC 10231, SC5314, *Δbgl2*, *Δmnn9*) were cultured on 6-well plates (Corning, Glendale, AZ, USA) or 96-well microplates (Cellvis, Mountain View, CA, USA). Yeast cells from stationary-phase cultures were washed with PBS and resuspended in RPMI 1640 medium (Gibco, Thermo Fisher Scientific, USA) at a final concentration of 3 × 10⁷ cells/ml (6-well plates) or 1 × 10^7^ cells/ml (96-well plates). Biofilms were incubated at 37°C with 5% CO_2_ for 90 minutes to allow initial adhesion. Non-adherent cells were removed by washing with PBS, and biofilms were further cultured for 6, 24, or 48 hours. The same protocol was used for the *Δefg1/Δcph1* strain culture.

### 2.3 Neutrophil isolation

Neutrophils were isolated from whole blood samples (EDTA-treated), obtained from healthy, anonymous donors through the Regional Blood Donation Center in Cracow, Poland, adhering to all ethical standards and confidentiality protocols. Blood samples were centrifuged at 300 x g for 20 minutes to separate the plasma. Two-thirds of the upper phase was discarded, and the remaining suspension was diluted with PBS (Biowest, Nuaille, France). The diluted suspension was layered onto a lymphocyte separation medium (Biowest, Nuaille, France) and centrifuged at 300 x g for 30 minutes. The low-density fractions were discarded, and the phase containing red blood cells (RBCs) and granulocytes was collected. RBCs were separated from granulocytes by incubation with 1% polyvinyl alcohol for 20 minutes. The granulocyte-containing upper layer was transferred to a new tube and centrifuged at 420 x g for 5 minutes. Remaining RBCs were lysed using 1 ml of Red Blood Lysis Buffer (Roche, Penzberg, Germany). The granulocyte pellet was resuspended in 1 ml of RPMI 1640 medium. Neutrophil purity was assessed by forward- and side-scatter flow cytometry, typically achieving >95% purity, and confirmed by analysis of CD11b and CD66b surface marker expression. After neutrophil isolation, cell viability was assessed using the Trypan Blue method. Typically, the isolated neutrophil population exhibited greater than 95% viability.

### 2.4 Collection and purification of supernatants from 48-hour *C. albicans* biofilms

*C. albicans* biofilms (strains ATCC 10231 and SC5314) were grown for 48 hours. The collected supernatants were centrifuged (4,000 × g, 10 min, 4°C) and purified using Ultrafree-CL Centrifugal Filter Units with a 0.22 µm membrane (Merck) to remove fungal cells. The purified samples were stored at -80°C until further analysis.

### 2.5 Oxygen level comparison

In order to compare the oxygen level in *C. albicans* biofilms at different development stages (6, 24 and 48 hours), the environmental probe Pt(II) meso-tetra(pentafluorophenyl)porphyrin (Frontier Scientific) was added to the culture medium at a final concentration of 1 µM. The Pt(II) porphyrin-based probe operates on the principle of dynamic fluorescence quenching by molecular oxygen. As a result, the fluorescent signal is suppressed in proportion to the oxygen concentration in the sample. Incubation with the probe was carried out at 37°C, 5% CO_2_ for 60 minutes, which allowed for its penetration into the biofilm structure. The fluorescence level was measured using a microplate reader (Synergy H1, BioTek) at λ excitation = 400 nm and λ emission = 655 nm, using the “area scanning” function.

### 2.6 Detection of hypoxia in neutrophils

Freshly isolated neutrophils were stained with Image-iT™ Green Hypoxia Reagent (Invitrogen, Waltham, Massachusetts, USA; Cat. #I14833; final concentration 5 µM) before being added to the biofilms and washed with PBS. Neutrophils (10^7^/ml in 100 µl RPMI medium) were then added to 6-, 24- or 48-hours *C. albicans* biofilms and incubated for 3 hours. Cells were imaged using an Olympus IX73 microscope in the FITC channel.

### 2.7 Apoptosis activation assay

Prior to addition to the biofilm, neutrophils were stained with the CellEvent™ Caspase-3/7 Detection Reagents kit (Invitrogen, Waltham, MA; cat. #C10423; final concentration 2 µM), according to the manufacturer’s instructions, and washed three times with PBS. Then, cells were added to biofilms, incubated for 3, 6 or 24 h and after incubation a series of microscopic images (FITC channel) were taken using an Olympus IX73 microscope. Fluorescence was quantified using Olympus cellSens Dimension 3.2 imaging software.

### 2.8 Measurement of MIP-1β and IL-8 production

Neutrophils were incubated with *C. albicans* biofilms for 24 hours. Following incubation, supernatants were collected by centrifugation at 500 × g for 10 minutes to remove cellular debris. The ELISA assay was performed according to the manufacturer’s instructions. MIP-1β levels in supernatants were quantified using the Human MIP-1β/CCL4 ELISA Kit (Sigma-Aldrich, Saint Louis, Missouri, USA; cat. #RAB0075). IL-8 levels were quantified using the BD OptEIA™ Human IL-8 ELISA Set (BD Biosciences, San Jose, California, USA; cat. #555244).

### 2.9 Immunofluorescent staining of HIF-1α

Immunofluorescent staining of HIF-1α was performed after 3 hours of incubation with biofilms. Neutrophils were fixed in 4% paraformaldehyde and permeabilized with 0.05% Triton X-100. The cells were then incubated overnight at 4°C with rabbit anti-HIF-1α antibodies (1:500; Cell Signaling Technology; Cat. #36169), followed by a 1-hour incubation at 37°C with Alexa Fluor 555-conjugated secondary anti-rabbit antibodies (Abcam, Cambridge, UK, Cat. No. ab150074; 1:500). The samples were subsequently imaged using an Olympus IX73 fluorescence microscope.

### 2.10 NETs visualization and quantification

Neutrophils (10^7^ cells/ml) were added to *C. albicans* biofilms formed on 96-well microplates in 100 µl of RPMI. Incubation was carried out for 1–24 hours at 37°C with 5% CO_2_. Neutrophils maintained in RPMI medium without exposure to biofilm served as the negative control. Post-stimulation, neutrophils were stained with Sytox Green (Invitrogen, Thermo Fisher Scientific, Waltham, MA, USA; Cat. #S7020; final concentration 1 µM), and the released NETs were visualized using a fluorescence microscope (IX73, Olympus). Additionally, cells were fixed with 4% paraformaldehyde, and NETs protein markers were labeled using rabbit anti-histone H3 (citrulline R2 + R8 + R17) antibody at 1:500 dilution (Abcam, Cambridge, UK; cat. #ab5103) or rabbit anti-neutrophil elastase (Abcam, Cambridge, UK; cat. #ab131260) with overnight incubation at 4°C. Then, secondary anti-rabbit antibodies (Alexa Fluor 555) at a 1:500 dilution were incubated for 1 hour at 37°C, and the samples were visualized using fluorescence microscopy.

### 2.11 Analysis of ROS production

Freshly isolated neutrophils were stained with dihydrorhodamine 123 (DHR 123; Invitrogen, Waltham, MA; Cat. #D23806; final concentration 5 μM), washed three times with PBS and then added to *C. albicans* biofilms. Neutrophils stimulated by PMA (25 nM) were used as the positive control, and cells in RPMI medium served as a negative control. The fluorescence of oxidized rhodamine 123 was measured (λ ex: 488 nm, λ em: 525 nm) using a microplate reader (H1, Biotek).

### 2.12 Western blot analysis

Neutrophils (3 × 10⁷/ml) were incubated with *C. albicans* biofilms formed for 48-hour on 6-well microplates. After 4 hours of incubation, neutrophils were lysed using cold RIPA buffer (10 mM Tris-HCl, pH 8.0; 1 mM EDTA; 0.5 mM EGTA; 1% Triton X-100; 0.1% Sodium Deoxycholate; 0.1% SDS; 140 mM NaCl) supplemented with protease and phosphatase inhibitors (Merck, Darmstadt, Germany). The samples underwent multiple freeze-thaw cycles in liquid nitrogen to ensure complete cell lysis and were then centrifuged at maximum speed. Protein concentrations were determined using the Pierce™ BCA Protein Assay Kit (Thermo Fisher Scientific, Waltham, MA, USA; cat. # 23225). Equal amounts of total protein (10 µg) were mixed with Laemmli sample buffer, heated at 95°C for 5 minutes, loaded onto 8% polyacrylamide gels, and subjected to SDS-PAGE electrophoresis (28). Proteins were transferred to PVDF membranes using a wet electroblotting system (BioRad, Hercules, CA, USA). Membranes were blocked with 5% non-fat milk in TBST for 1 hour and incubated overnight at 4°C with primary antibodies anti-Mcl-1 (1:1000, Abcam, Cambridge, UK; cat. #94296) and HIF-1α (1:1000; Cell Signaling Technology, Danvers, Massachusetts, USA, cat. #36169). β-actin was selected as a protein loading control. For β-actin detection mouse anti-β-actin primary antibodies (1:10000, Cell Signaling Technology, Danvers, Massachusetts, USA; cat. #3700) were used. Then membranes were incubated with HRP-conjugated secondary antibodies (anti-rabbit: 1:1000; anti-mouse 1:10000; R&D Systems, Minnesota, USA; cat. #HAF007) at room temperature for 1 hour, and protein bands were visualized using a ChemiDoc Imaging System (BioRad) after the addition of Westar Supernova Substrate (Cyanagen, Bologna, Italy; cat. #XLS3L). Bands intensities were quantified using ImageJ software and normalized to the loading control.

### 2.13 Neutrophil elastase activity test

Neutrophils were incubated with *C. albicans* biofilms for up to 3 hours, after which the supernatants were carefully collected to ensure minimal disruption of the biofilm structure and centrifuged to remove any residual cells or debris. Neutrophil elastase activity in the clarified supernatants was then quantified using the fluorometric Neutrophil Elastase Activity Assay Kit (Sigma-Aldrich, Saint Louis, Missouri, USA; cat. #MAK246) according to the manufacturer’s instructions.

### 2.14 HIF-1α inhibition

Neutrophils were preincubated for 30 minutes with LW6 (MedChemExpress, Monmouth Junction, USA; cat. #HY-13671; 2 µM) and PX478 (MedChemExpress, Monmouth Junction, USA; cat. #HY-10231, 10 µM). The cells were then washed and added to *C. albicans* biofilms. Then the effect of HIF-1α inhibitors on ROS and NETosis was analyzed, after 1 hour or 6 hours, respectively.

### 2.15 Statistical analysis

Statistical analysis was performed using GraphPad Prism 10.0 software (GraphPad, CA, USA). Results are presented as the mean ± standard error of the mean (SEM). Comparisons between two groups were assessed using an unpaired t-test, while differences among multiple groups were analyzed using one-way ANOVA followed by Dunnett’s *post-hoc* test. A p-value < 0.05 was considered statistically significant (* p < 0.05, ** p < 0.01, *** p < 0.001, **** p < 0.0001), while p > 0.05 was considered not significant (ns). Each experiment was repeated at least five times using samples from different donors to ensure reproducibility of the results.

## 3 Results

### 3.1 Contact with *C. albicans* biofilm induces hypoxia in neutrophils

**Figure 1:**
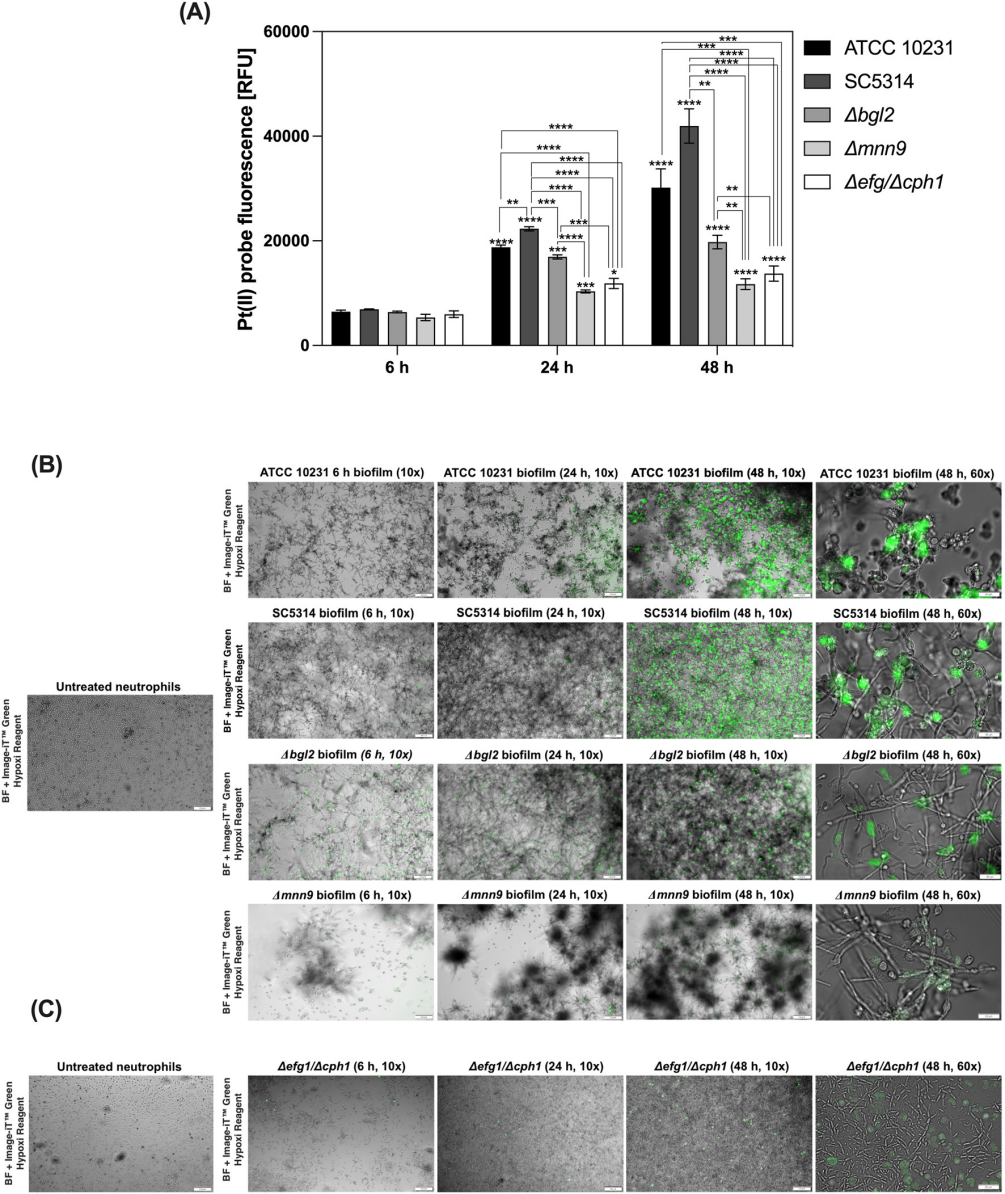
Oxygen depletion in *C. albicans* biofilms and induction of intracellular hypoxia in neutrophils. **(A)** *C. albicans* biofilms (strains ATCC 10231, SC5314, Δbgl2, Δmnn9, Δefg1/Δcph1) were cultured in 96-well plates for 6, 24, or 48 hours. To assess oxygen levels, Pt(II) meso-tetra(pentafluorophenyl) porphyrin was added to the medium at a final concentration of 1 μM and incubated for one hour. Fluorescence intensity of probe was measured using a microplate reader (excitation/emission: 400/655 nm). A representative result from three independent experiments is shown. Statistical significance was first evaluated for each strain between different biofilm growth time points, followed by comparisons between strains within the same time point. Results were considered statistically significant at p < 0.05 (*p < 0.05; **p < 0.01; ***p < 0.001; ****p < 0.0001), p > 0.05 – not significant (ns). For clarity, ns differences (p > 0.05) are not marked in the figure. **(B)** Representative fluorescence microscopy images showing induction of intracellular hypoxia in neutrophils after exposure to *C. albicans* biofilms. Neutrophils were stained with Image-iT™ Green Hypoxia Reagent after 2 h of co-culture with C. albicans biofilms formed by ATCC 10231, SC5314, *Δbgl2*, *Δmnn9* (6, 24, and 48 h), and **(C)** *Δefg1/Δcph1* strain, which does not form mature biofilms, and was used as a control for planktonic cells. Neutrophils not exposed to biofilms (untreated neutrophils) served as the negative control. The images were taken at two magnifications: 10× and 60× as indicated. The biofilm image was captured using bright-field (BF) microscopy.

Oxygen availability within biofilms is a critical factor influencing microbial physiology and host immune responses. Intensive microbial growth and high metabolic activity within these structures often lead to a significant reduction in oxygen levels in the infection microenvironment (24, 29). In fungal communities, such hypoxic conditions can modulate pathogen survival strategies and impact the functionality of infiltrating immune cells. To investigate oxygen consumption dynamics during *C. albicans* biofilm development *in vitro*, we measured oxygen levels at different stages of biofilm maturation in selected *C. albicans* strains and evaluated the impact of these conditions, particularly hypoxia, on neutrophil responses. The analysis was performed on *C. albicans* wild-type strains ATCC 10231 and SC5314. ATCC 10231 is typically considered less virulent, while SC5314, a bloodstream isolate, is known for its higher pathogenicity and unique morphological characteristics. Additionally, we used mutants with defects in cell wall polysaccharide synthesis - *Δbgl2* (impaired β-1,3-glucan synthesis) and *Δmnn9* (defective mannan biosynthesis), as well as the *Δefg1/Δcph1* strain, which lacks the ability to form biofilms and remains in a planktonic state. To assess oxygen levels within biofilms, we used an oxygen-sensitive probe [Pt(II) meso-tetra(pentafluorophenyl)porphine] that undergoes fluorescence signal quenching in response to oxygen availability. Using this approach, we compared oxygenation profiles across different *C. albicans* strains (**Figure 1A**). At the 6-hour time point, no significant differences in oxygen levels were observed between strains. After 24 and 48 hours, the lowest oxygen levels were detected in wild-type biofilms, with SC5314 exhibiting the most pronounced depletion, followed by ATCC 10231. The *Δbgl2* mutant showed slightly higher oxygen availability than the wild-type strains, while *Δmnn9* displayed the highest oxygen levels among biofilm-forming strains. The *Δefg1/Δcph1* strain, which remains planktonic, showed fluorescence levels similar to *Δmnn9*.

Our results indicate that neutrophils are partially able to penetrate into the deeper layers of the biofilm (**Supplementary Figure 1**). The direct contact between neutrophils and the biofilm suggests that they are increasingly exposed to specific conditions, which may shape their functional responses. To investigate how the gradual development of hypoxic conditions affects neutrophils, we employed the Image-iT™ Green Hypoxia Reagent, a fluorescent intracellular oxygen sensor specifically designed for live-cell imaging. This probe remains non-fluorescent in normoxic conditions but emits a robust green fluorescence in hypoxia, allowing precise visualization of hypoxic states in cells. Neutrophils were stained with this probe prior to exposure to biofilms formed by selected strains. To investigate the dynamics of hypoxia induction, we incubated neutrophils with biofilms at various developmental stages, assessing the influence of biofilm maturation and density on hypoxia development in these immune cells. As a control, neutrophils maintained under normoxic conditions without biofilm stimulation were used. Our results indicated that biofilm development by wild-type strains significantly influences hypoxia induction in neutrophils (**Figure 1B**). Neutrophils in contact with late-stage biofilms (48 hours) displayed significantly elevated fluorescence, correlating with pronounced hypoxia, compared to those exposed to maturing biofilms (6–24 hours). Notably, hypoxic response was detectable as early as 2 hours post-exposure, highlighting the rapid impact of biofilm-generated conditions on neutrophil physiology. In contrast, neutrophils without biofilm contact exhibited low fluorescence levels, confirming their normoxic state under experimental conditions. The analysis of hypoxic responses in neutrophils exposed to biofilms formed by mutant strains revealed significant differences, with the weakest hypoxic response observed in neutrophils exposed to the *Δmnn9* mutant. It is worth noting that biofilms formed by *Δmnn9* were notably less homogeneous, characterized by dispersed cell clusters with weak adhesion, which diffused into the surrounding medium (visible as gray patches in microscopic images). In contrast, the *Δbgl2* biofilm exhibited a morphology similar to that of wild-type biofilms and showed only a slightly reduced capacity to induce hypoxia in neutrophils. These findings suggest that differences in ECM composition, particularly the disruption of mannan biosynthesis in *Δmnn9*, may influence biofilm architecture and oxygen diffusion within the biofilm. Neutrophils exposed to the *Δefg1/Δcph1* strain did not exhibit significant hypoxia (**Figure 1C**). Since this strain remains in a planktonic state and does not form biofilm, the lack of ECM components and weaker metabolic activity (**Supplementary Figure 2**) likely prevent oxygen depletion and consequently the induction of neutrophil hypoxia. A control experiment was also conducted, in which neutrophils were incubated with the supernatant collected from biofilms. Under these conditions, no induction of hypoxia was detected in neutrophils, suggesting that the observed hypoxic response is associated with direct interaction with biofilm environment (**Supplementary Figure 3**).

### 3.2 Contact with *C. albicans* biofilm prolongs neutrophil lifespan through the accumulation of Mcl-1

**Figure 2:**
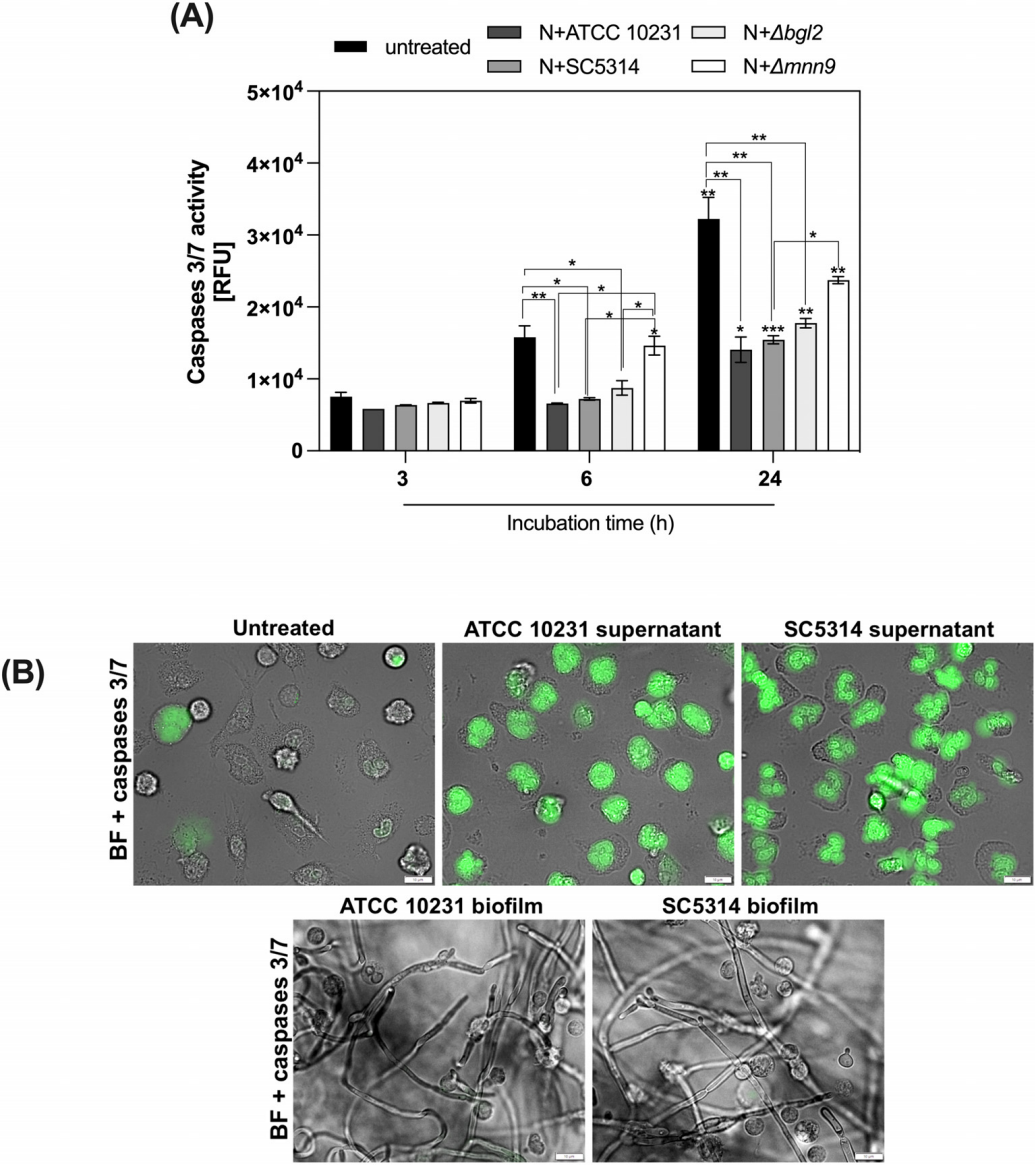
Modulation of neutrophils survival by *C albicans* biofilms. **(A)** 48-hour *C albicans* biofilms (strains ATCC 10231, SC5314, *Δmnn9*, *Δbgl2*) were incubated with neutrophils for 3, 6, and 24 hours. Before being added to the biofilm, the cells were labeled with the CellEvent Caspases 3/7 kit. A series of microscopic images were then taken for each time point, and the mean fluorescence intensity was measured using the CellSens software (Olympus). Statistical analysis was performed to assess changes in caspase 3/7 activity over time within each strain (asterisks above bars) and differences between strains at each time point (asterisks connecting bars). The results were considered statistically significant for p < 0.05 (*p < 0.05, **p < 0.01, ***p < 0.001) and ns, p > 0.05. For clarity, ns differences (p > 0.05) are not marked in the figure. The experiment was repeated five times **(B)**. Representative microscopic images showing caspase 3/7 activity in control cells and those incubated with supernatants or biofilms (ATCC 10231 and SC5314) for 6 hours. Images were acquired in brightfield (BF) and FITC channels using an Olympus IX73 microscope.

Since neutrophils are terminally differentiated cells characterized by short lifespan (30), we examined whether hypoxic conditions may affect their survival upon contact with *C. albicans* biofilms. To this end, neutrophils were treated with a fluorogenic substrate specific for caspases 3/7 (DEVD) and subsequently incubated with 48-hour biofilms formed by various *C. albicans* strains (ATCC 10231, SC5314, *Δmnn9*, *Δbgl2*) for 3, 6, and 24 hours. The strain *Δefg1/Δcph1* was eliminated from consideration due to a lack of hypoxic conditions. The The data, expressed as relative fluorescence units (RFU) representing caspase 3/7 activity, revealed strain- and time-dependent differences in apoptosis and are shown in **Figure 2A**. In untreated neutrophils, caspase 3/7 activity increased progressively over time, reaching the highest levels at 24 hours, reflecting the natural progression of apoptosis. In contrast, neutrophils exposed to biofilms exhibited a marked reduction in caspase activity, with this effect being particularly evident after 24 hours of incubation. Only *Δmnn9* biofilms had a weaker apoptosis-inhibiting effect. To determine whether the observed inhibition of apoptosis was due to direct contact with the biofilm microenvironment or to soluble factors released by *C. albicans*, neutrophils were incubated with supernatants collected from 48-hour biofilms of ATCC 10231 and SC5314 strains. Supernatants were filtered (0.22 μm) to remove cellular debris before exposure. Interestingly, biofilm-derived supernatants strongly induced caspase 3/7 activation, whereas direct contact with biofilms suppressed this enzymatic activity in neutrophils. Representative microscopy images illustrating differences in caspase 3/7 activity among control cells, neutrophils incubated with biofilm supernatants, and those exposed to biofilms are shown in **Figure 2B**. These results suggest that the balance between pro-apoptotic signals and survival mechanisms is dynamically regulated by the biofilm microenvironment. This finding also indicates the possible involvement of hypoxia-adaptive signaling pathways that promote neutrophil survival.

**Figure 3:**
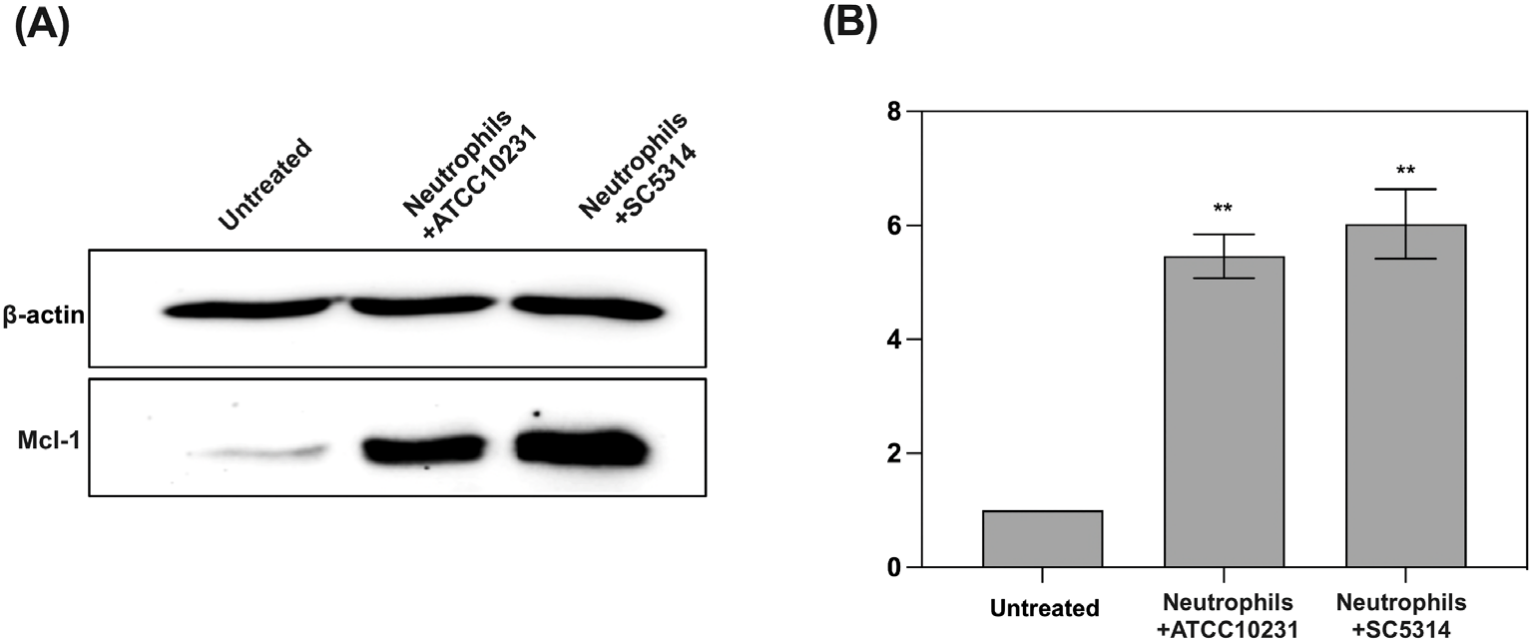
Analysis of anti-apoptotic protein Mcl-1 accumulation in response to contact with *C albicans* biofilms (wild-type strains). **(A)** Representative Western blot result showing Mcl-1 levels in neutrophils after 3 hours of co-culture with 48-hour *C albicans* biofilms (ATCC 10231 and SC5314). For Mcl-1 analysis primary, rabbit antibodies anti-Mcl-1 (1:1000, Cell Signaling) and secondary HRP-conjugated antibodies (1:1000; R&D) were used. Antibodies anti-β-actin (1:10000, Cell Signaling) were used as a protein loading control. **(B)** Densitometric analysis was performed using ImageJ software, and the results are presented as relative Mcl-1 levels in neutrophils. The results were considered statistically significant for p < 0.05 (**p < 0.01). Data represent the results of three independent biological replicates.

Neutrophil apoptosis may also be influenced by biofilm-derived soluble factors, not only by the biofilm environment. To determine whether the observed inhibition of apoptosis was due to direct contact with biofilm microenvironment or soluble factors released by *C. albicans*, neutrophils were incubated with supernatants collected from 48-hour biofilms of ATCC 10231 and SC5314 strains. Supernatants were filtered (0.22 μm) to remove cellular debris before exposure. Interestingly, biofilm-derived supernatants strongly induced caspase 3/7 activation, whereas direct contact with biofilms suppressed this enzymatic activity in neutrophils. These results suggest that the balance between pro-apoptotic signals and survival mechanisms is dynamically regulated by the biofilm microenvironment. This finding also indicates the possible involvement of hypoxia-adaptive signaling pathways that promote neutrophil survival. Representative microscopy images illustrating differences in caspase 3/7 activity among control cells, neutrophils incubated with biofilm supernatants, and those exposed to biofilms are shown in **Figure 3**.

To further investigate the mechanism by which *C. albicans* biofilms promote neutrophil survival, we analyzed the levels of Mcl-1, a key anti-apoptotic protein known to be involved in neutrophil survival (31). Western blot analysis indicated that neutrophils exposed to both ATCC 10231 and SC5314 biofilms exhibited significantly elevated levels of Mcl-1 compared to untreated neutrophils (**Figures 3A, B**). This suggests that biofilm contact triggers the accumulation of Mcl-1, which in turn delays apoptosis and prolongs the lifespan of neutrophils.

**Figure 4:**
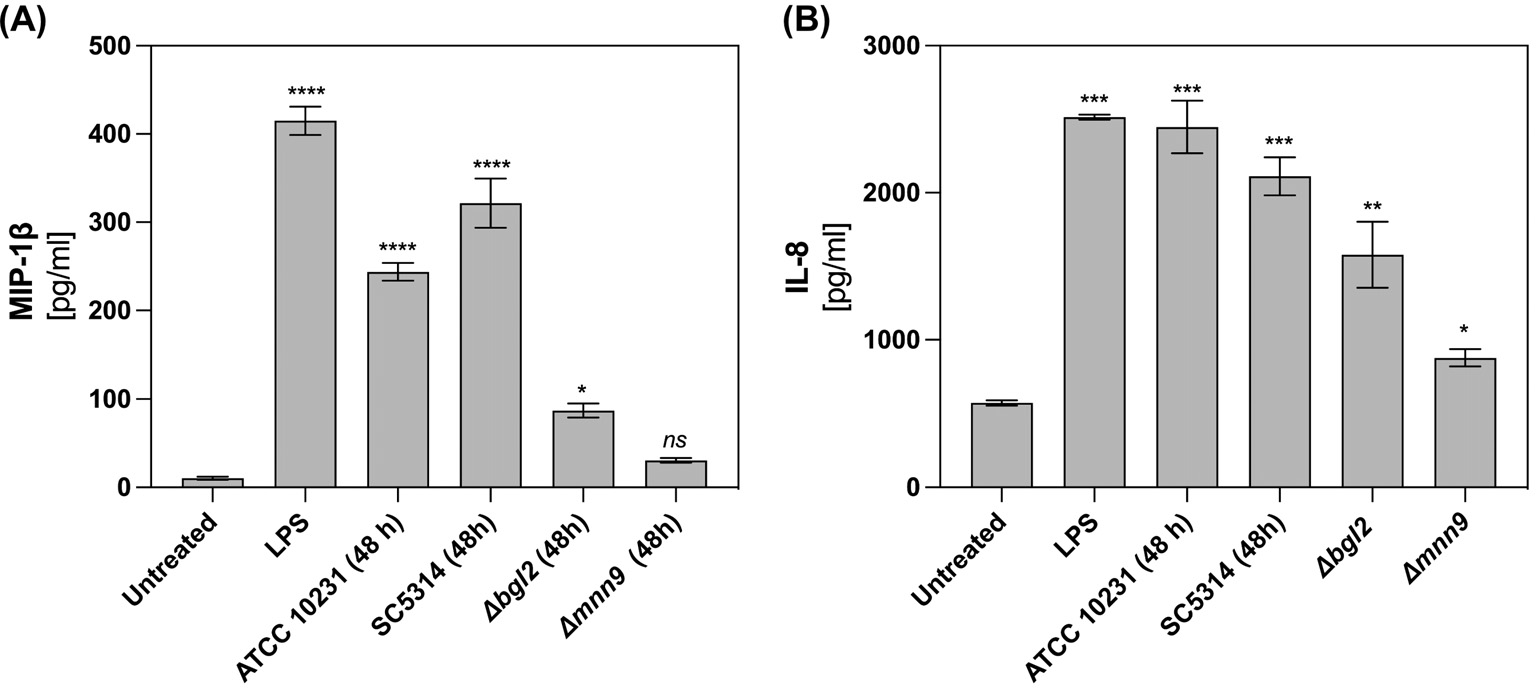
MIP-1β and IL-8 production in neutrophils stimulated by *C albicans* biofilms. For MIP-1β **(A)** and IL-8 **(B)** quantification neutrophils were incubated with *C albicans* biofilms (ATCC 10231, SC5314, *Δmnn9*, *Δbgl2*) for 24 h and chemokine level was quantified in supernatants using ELISA test. Data represent the mean ± SEM. The results were considered statistically significant for p < 0.05 (*p < 0.05, **p < 0.01, ***p < 0.001, ****p < 0.0001), ns, p > 0.05. The experiment was repeated five times independently.

Neutrophil survival can also be modulated by immune-derived factors at infection sites, particularly chemokines. We focused on MIP-1β (CCL4), a CC chemokine involved in neutrophil recruitment, activation, and survival, particularly under hypoxic conditions (25). Another key regulator is IL-8 (CXCL8), a CXC chemokine known for its chemotactic and pro-inflammatory roles, as well as its impact on neutrophil survival. To assess the secretion of IL-8 and MIP-1β by neutrophils, cells were incubated for 24 hours with mature (48-hour) biofilms formed by wild-type (*C. albicans* ATCC 10231 and SC5314) and ECM mutants (*Δmnn9* and *Δbgl2*). LPS stimulation served as a positive control for chemokine secretion. ELISA quantification (**Figure 4**) revealed that wild-type biofilms significantly enhanced MIP-1β (**Figure 4A**) and IL-8 (**Figure 4B**) secretion compared to unstimulated neutrophils. In contrast, exposure to *Δbgl2* biofilms resulted in reduced MIP-1β secretion and moderate IL-8 production. The *Δmnn9* mutant did not significantly alter MIP-1β levels relative to the negative control and induced only minimal IL-8 secretion. These findings indicate that while hypoxia is a key factor regulating neutrophil survival in the biofilm microenvironment, the secretion of chemokines such as IL-8 and MIP-1β may also contribute to shaping neutrophil lifespan and function during fungal infections.

### 3.3 Neutrophil contact with *C. albicans* biofilm affects the stabilization of HIF-1α

**Figure 5:**
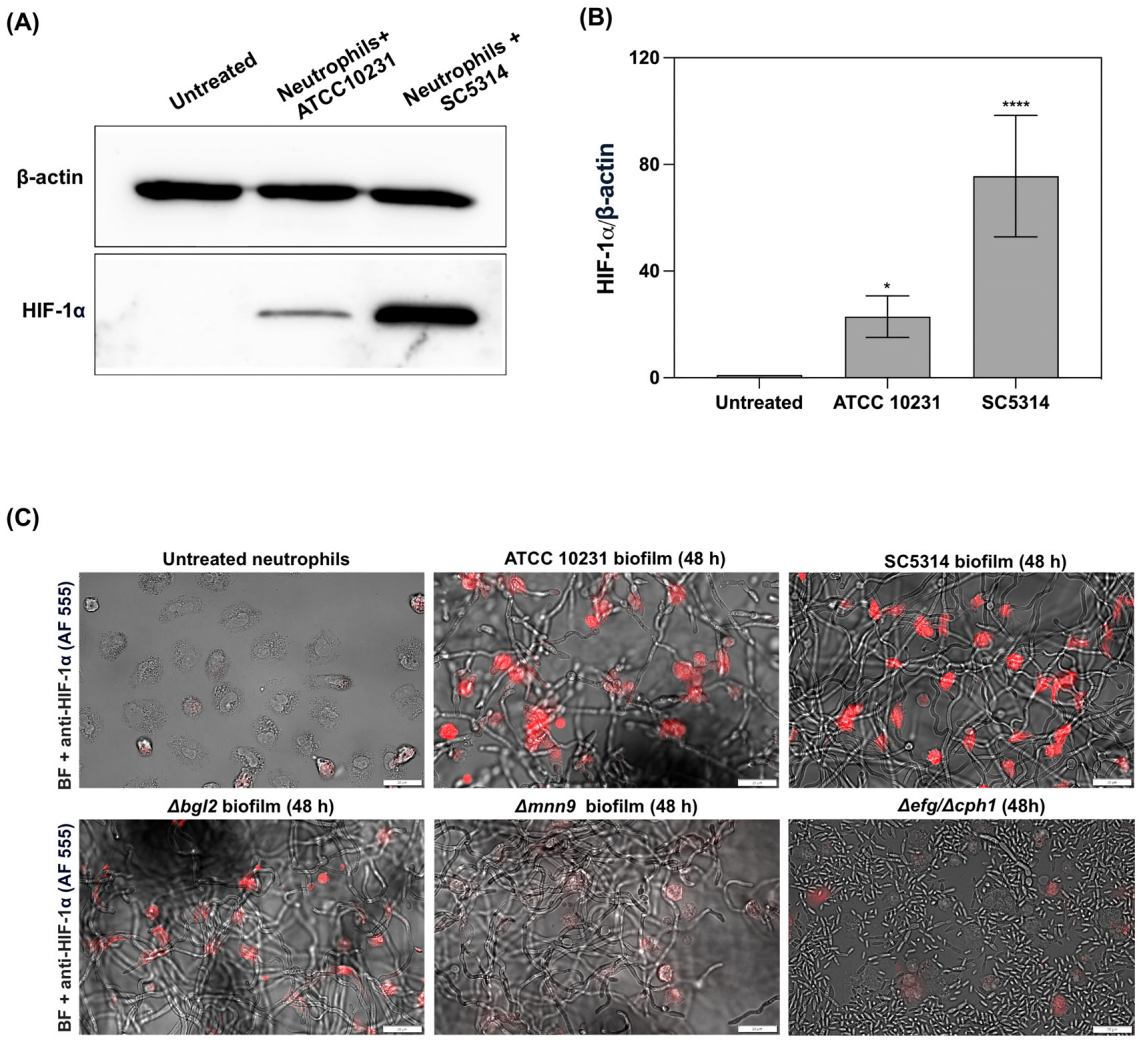
Stabilization of HIF-1α in neutrophils following exposure to *C albicans* biofilms. **(A)** Representative Western blot result showing HIF-1α levels in neutrophils after 3 hours of co-culture with 48-hour *C albicans* biofilms formed by the ATCC 10231 and SC5314 strains. For HIF-1α analysis primary, rabbit antibodies anti-HIF-1α (1:1000, Cell Signaling) and secondary HRP-conjugated secondary antibodies (1:1000; R&D) were used. β-actin (mouse antibodies anti- β-actin, 1:10000, Cell Signaling) was used as a loading control. **(B)** Densitometric analysis of Western blot was performed in ImageJ software and presented as the relative levels of HIF-1α in neutrophils. The results were considered statistically significant for p < 0.05 (*p <0.05, ****p <0.0001). Data represent the results of five independent biological replicates (n=5). **(C)** Immunofluorescence staining of HIF-1α (red channel, Alexa Fluor 555) in neutrophils co-cultured with *C albicans* biofilms (ATCC 10231, SC5314, *Δbgl2*, *Δmnn9* and *Δefg1/Δcph1*). The image was also acquired using bright-field (BF) microscopy.

The observed decrease in caspase activity and high levels of the anti-apoptotic protein Mcl-1 suggested that one of the factors regulating neutrophil physiology at later stages of biofilm interaction could be the transcription factor HIF-1α, which is known for its role in modulating neutrophil survival under hypoxic conditions (25). Given that the mature 48-hour biofilms generated the strongest hypoxic responses, we chose this time point for the subsequent experiments. Neutrophils were co-cultured for 3 hours with 48-hour *C. albicans* biofilms formed by the ATCC 10231 and SC5314 strains, and HIF-1α levels were analyzed in cell lysates using Western blot analysis. The results indicated that exposure to *C. albicans* biofilms leads to an increase in HIF-1α stabilization in neutrophils, with a markedly stronger effect observed in those co-cultured with the SC5314 strain (**Figure 5A**). Densitometry analysis (**Figure 5B**) further confirms this observation, showing a significant elevation in the relative levels of HIF-1α in neutrophils exposed to the SC5314 biofilm, with levels nearly doubling compared to those in the ATCC 10231 group. Untreated neutrophils exhibited negligible HIF-1α accumulation, suggesting that direct interaction with *C. albicans* biofilms is a critical factor in the stabilization of this transcription factor. Furthermore, immunofluorescence analysis (**Figure 5C**) provided visual confirmation of increased HIF-1α levels in neutrophils following exposure to *C. albicans* biofilms. To further elucidate the role of specific biofilm components in this process, we compared these results with neutrophils incubated with biofilms formed by the *Δbgl2* and *Δmnn9* mutants. The analysis demonstrated that while contact with *Δbgl2* biofilms led to a moderate increase in HIF-1α stabilization, and exposure to *Δmnn9* biofilms resulted in only marginal accumulation of this transcription factor. A similar lack of HIF-1α stabilization was observed in neutrophils incubated with the *Δefg1/Δcph1* mutant, which remains in a planktonic form. This further supports the hypothesis that biofilm structure plays a crucial role in inducing HIF-1α accumulation in neutrophils.

### 3.4 The development of *C. albicans* biofilm influences the change in the profile of immune response of neutrophils

**Figure 6:**
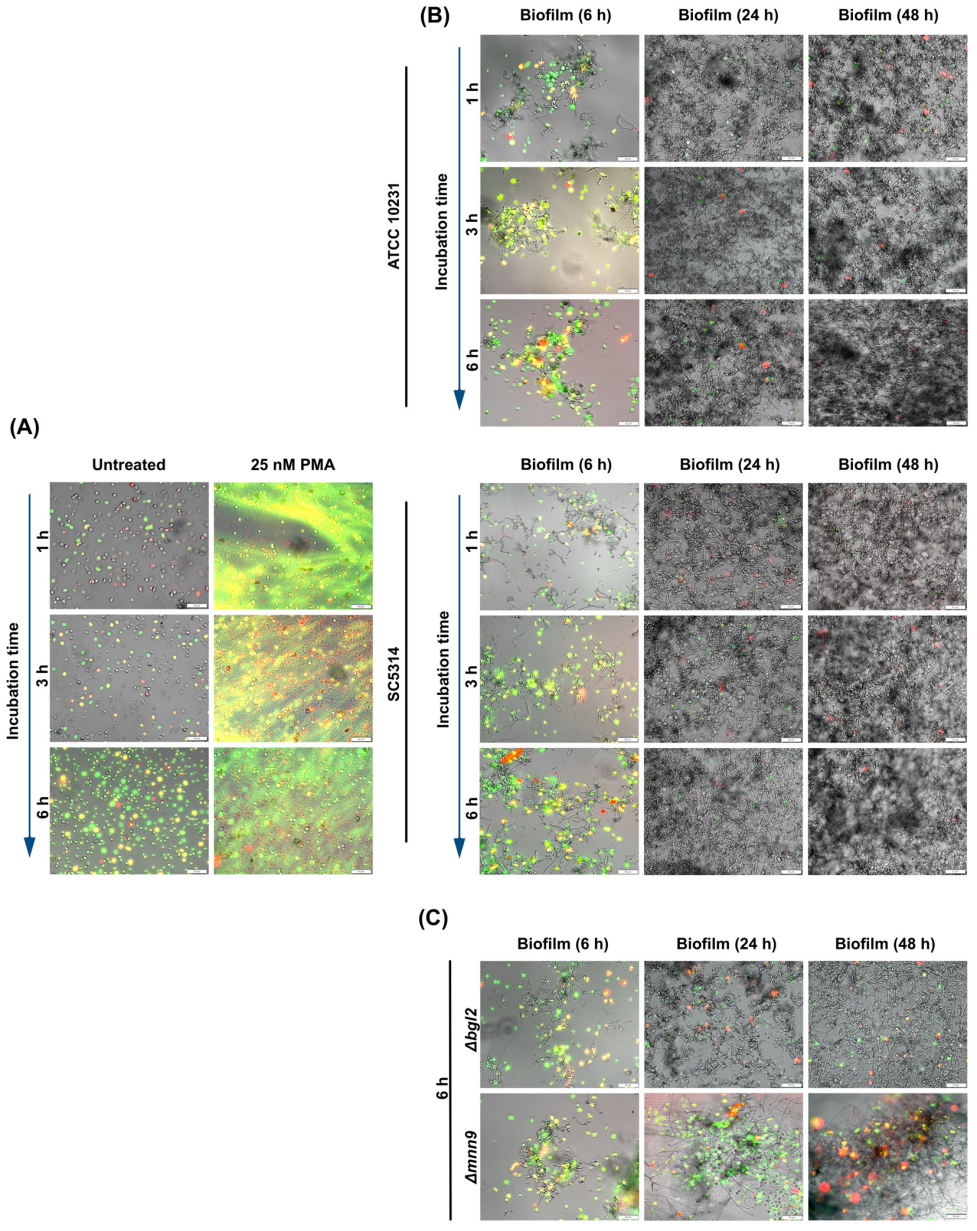
Analysis of NET formation in response to *C albicans* biofilm. **(A)** The panel presents control conditions, where neutrophils incubated in RPMI without biofilm contact served as a negative control, while PMA-stimulated neutrophils (25 nM) represented the positive control. **(B)** Neutrophils were incubated with *C albicans* biofilms formed by ATCC 10231 and SC5314 strains at different stages of biofilm development (6, 24, and 48 hours). NET formation was assessed using fluorescence microscopy with Sytox Green nucleic acid dye (green channel) and anti-citrullinated histone H3 antibodies (Alexa Fluor 555, red channel). Bright-field (BF) microscopy was used to visualize biofilm structure. **(C)** To evaluate the impact of ECM composition, neutrophils were incubated for 6 hours with biofilms formed by *C albicans* ECM mutants (*Δbgl2*, *Δmnn9*) at different biofilm developmental stages (6, 24 or 48-hours). NETs were detected using Sytox Green fluorescence (green channel) and anti-citrullinated histone H3 antibodies, followed by labeling with secondary anti-rabbit antibodies (Alexa Fluor 555, red channel). BF microscopy provided structural visualization of the biofilms. The experiment was repeated ten times independently using neutrophils from different donors.

To explore the downstream effects of increased hypoxia in neutrophils upon contact with *C. albicans* biofilm, we investigated the effect of biofilm development and the associated gradual decrease in oxygen concentration on the activation of main neutrophil killing strategies. The first step was to examine how the ability to activate NETosis evolves over time, as this mechanism, in its classical ROS-dependent form, requires NADPH oxidase activation and the presence of oxygen as a substrate for ROS production. NETosis activation within biofilms was assessed using Sytox Green, a dye that selectively binds to DNA released by neutrophils in the form of NETs. To ensure that the observed structures were typical NETs rather than DNA released from necrotic cells or biofilm-derived nucleic acids, we assessed the colocalization of two characteristic markers of NETs - citrullinated histone H3 (**Figure 6**) and neutrophil elastase (NE) (**Supplementary Figure 4**). **Figure 6** shows the merged images (mCherry + FITC channel). Additional images showing Sytox Green staining alone at lower magnification, without antibody labeling, are provided in the **Supplementary Figure 5**. The NETosis analysis was conducted over varying incubation times (1, 3, and 6 hours) with biofilms at different stages of development (6, 24, and 48 hours), allowing us to evaluate the correlation between biofilm-induced hypoxia and NET formation. As a negative control, neutrophils incubated in RPMI without biofilm contact were used, while neutrophils stimulated with phorbol 12-myristate 13-acetate (PMA) served as a positive control (**Figure 6A**). In neutrophils exposed to the 6-hour biofilm of ATCC 10231 and SC5314, we observed a time-dependent increase in Sytox Green fluorescence. This was accompanied by the progressive appearance of citrullinated histone H3 (**Figure 6B**) and neutrophil elastase (**Supplementary Figure 4**), indicating active NETosis process. However, in the case of more developed biofilms (24h and 48h), NET formation was significantly inhibited. This pattern was consistent across both wild-type *C. albicans* strains. To investigate whether structural changes in the biofilm ECM affect NETosis activation, we conducted analyses using biofilms formed by *C. albicans* mutants: *Δbgl2* and *Δmnn9*. In this case, the assessment focused on neutrophil responses after 6 hours of incubation, as this time point was selected to capture the maximum effect of NETosis activation. The results (**Figure 6C**) showed that biofilms formed by the *Δbgl2* mutant exhibited a similar ability to inhibit NETosis compared to wild-type strains. In contrast, *Δmnn9* biofilms consistently induced a strong NETosis response at every stage of development. This finding suggested that the presence of the mannan layer and its influence on the formation of a hypoxic microenvironment by restricting oxygen diffusion may play a significant role in modulating this response mechanism.

**Figure 7:**
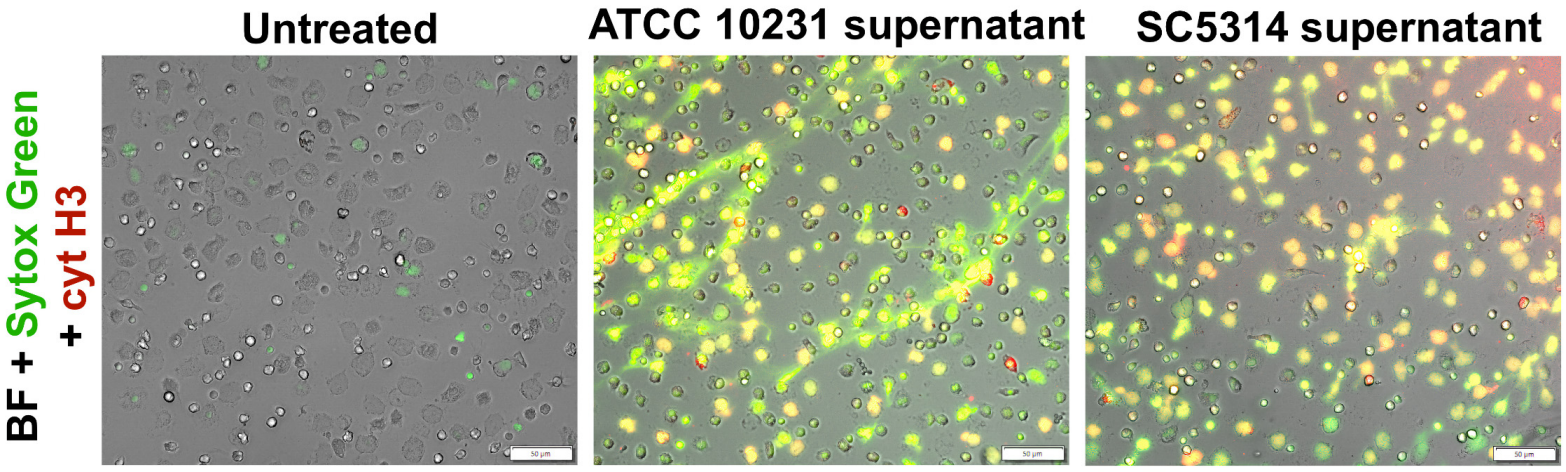
Analysis of NETosis activation in response to supernatants from ATCC 10231 and SC5314 biofilms. Neutrophils were incubated with 100 μl of supernatants collected from 48-hour ATCC 10231 and SC5314 biofilms for 3 hours. NETs were stained using Sytox Green and anti-citrullinated histone H3 antibodies, followed by labeling with secondary anti-rabbit antibodies (Alexa Fluor 555, red channel). Cells was visualized with an Olympus IX73 fluorescence microscope.

Analogous to the neutrophil viability assays, we determined whether direct contact with the biofilm structure and its microenvironment is the primary factor suppressing NETosis or if this process is driven by soluble factors released by *C. albicans* into the supernatant. The results (**Figure 7**) revealed that supernatants significantly enhanced NETosis levels compared to both the control and neutrophils directly interacting with the biofilm. These findings suggest that while various factors released by *C. albicans* play a crucial role in NETosis induction, as the infection progresses and the biofilm matures, its denser structure may lead to a reduction in NETosis activation, potentially due to changes in the biofilm architecture and oxygen accessibility.

**Figure 8:**
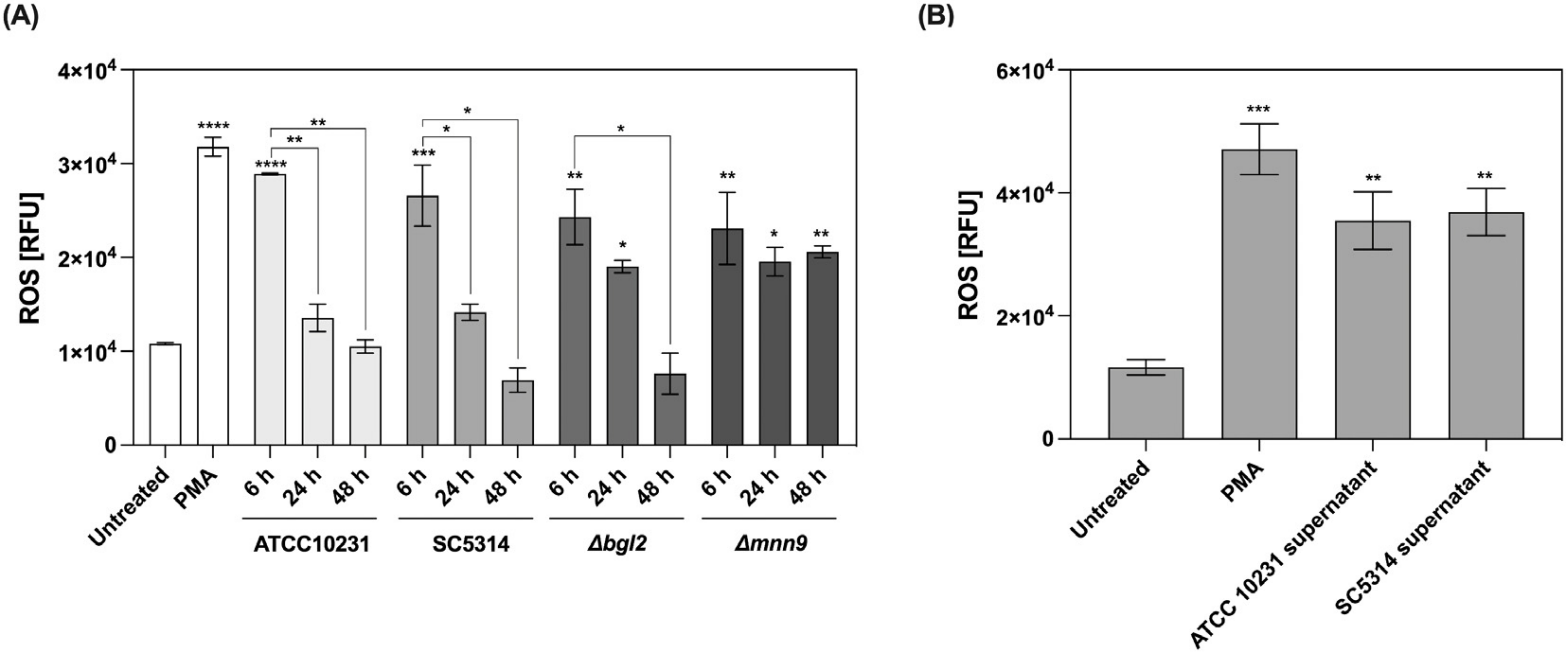
Measurement of reactive oxygen species (ROS) production in neutrophils exposed to *C albicans* biofilms and their supernatants. **(A)** Neutrophils were stained with DHR123 (final concentration 5 μM) before being added to the 48-hour biofilm (ATCC 10231, SC5314, *Δbgl2*, and *Δmnn9*), then incubated for 1 (h) ROS levels were quantified using a microplate reader. Asterisks above individual bars indicate statistically significant differences in ROS levels compared to the untreated control. Asterisks connecting the bars represent statistical comparisons within each strain, highlighting changes in ROS production at different stages of biofilm development. The experiment was repeated eight times independently. **(B)** Additionally, the effect of biofilm supernatants collected from 48-hours biofilms (ATCC 10231 and SC5314) on ROS production was assessed. Statistical analysis compared ROS production in neutrophils exposed to biofilms or their supernatants with the unstimulated control. The experiment was repeated five times independently. The results were considered statistically significant for p < 0.05 (*p < 0.05, **p < 0.01, ***p < 0.001, ****p < 0.0001), ns, p > 0.05. For clarity, ns differences (p > 0.05) are not marked in the figure.

To further explore the impact of interaction with *C. albicans* biofilm on neutrophil function, we measured the production of ROS, a critical component of the neutrophil’s antimicrobial arsenal and a key trigger of NETosis (**Figure 8A**). Since ROS generation is a relatively rapid process, measurements were performed after 1 hour of neutrophil incubation with 48-hour biofilms. For this purpose, neutrophils were pre-stained with the dihydrorhodamine 123 (DHR123), a sensitive intracellular ROS indicator. Untreated neutrophils cultured in RPMI medium served as the negative control. For the positive control, PMA—a potent activator of protein kinase C—was used to confirm the maximum ROS production capacity of neutrophils. The obtained results demonstrated that as the biofilm matures, its ability to induce ROS production in neutrophils gradually decreases. In the case of wild-type strains, high ROS levels were observed only in 6-hour biofilms. In contrast, in the presence of mature (24- and 48-hour) biofilms formed by ATCC 10231 and SC5314 strains, ROS levels did not exceed the baseline response observed in the negative control. Early-stage (6-hour) biofilms formed by the *Δbgl2* mutant induced a ROS response comparable to that of wild-type strains; however, in 24-hour biofilms, ROS levels exceeded those recorded in the control and in biofilms of ATCC 10231 and SC5314. Notably, a strong inhibitory effect on ROS production was not observed in the case of the *Δmnn9* mutant. At all biofilm development stages (6, 24, and 48 hours), biofilms formed by the *Δmnn9* strain induced a consistent ROS response, maintaining approximately 60% of the levels observed in the positive control. Additionally, ROS production was assessed in response to supernatants collected from 48-hour biofilms formed by ATCC 10231 and SC5314 strains. The results (**Figure 8B**) demonstrated a significant increase in ROS levels compared to the control, aligning with previous findings indicating that soluble factors released by *C. albicans* biofilms do not exert a pronounced inhibitory effect on neutrophil activity but rather contribute to its activation.

**Figure 9:**
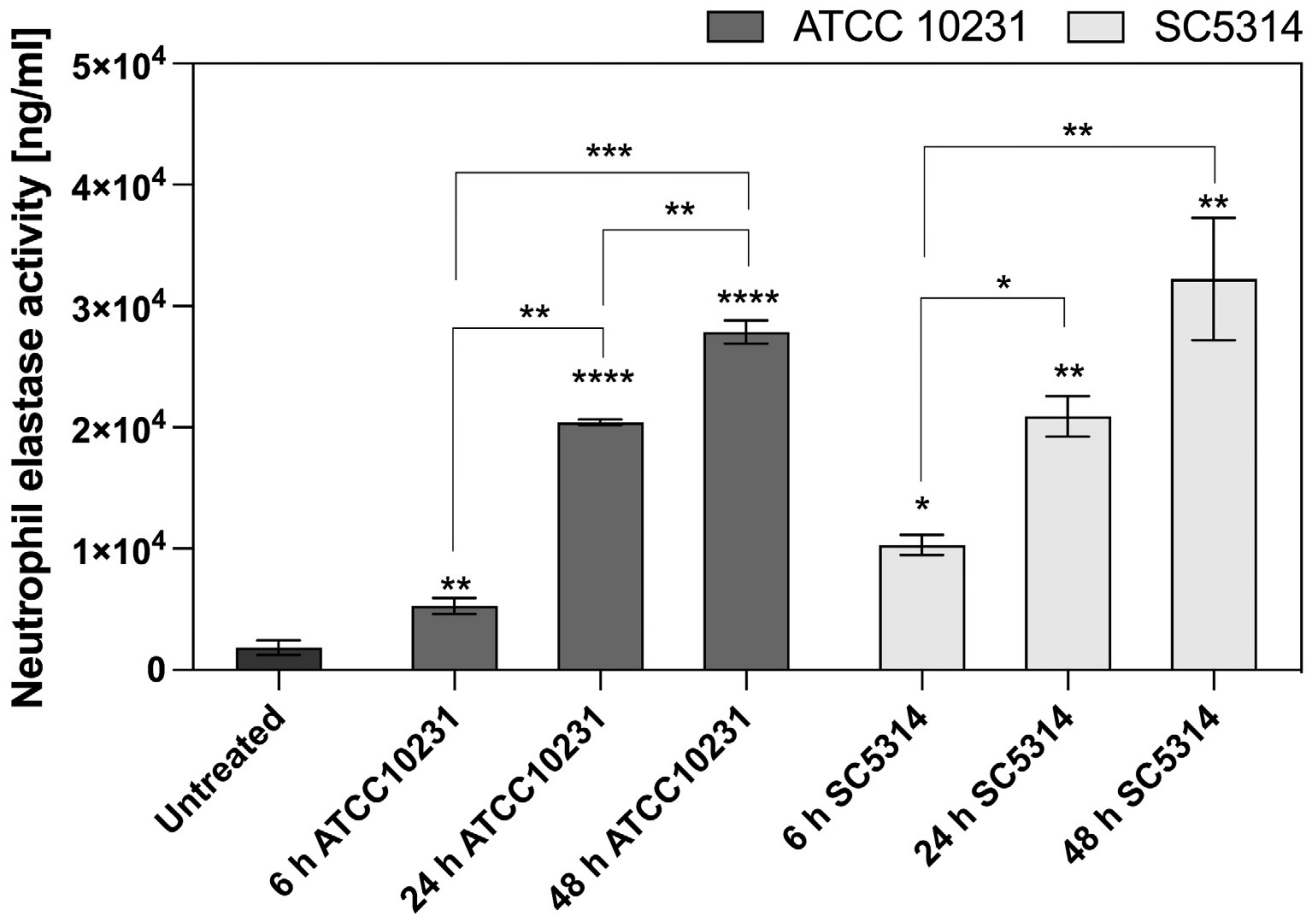
Neutrophil elastase activity after contact with *C. albicans* biofilm. Neutrophil elastase activity was assayed after 3 h of incubation of neutrophils with 6-, 24-, 48-hour *C. albicans* biofilms (ATCC 10231, SC5314) using the Neutrophil Elastase Activity Assay Kit (Merck). Statistical comparisons were performed to assess neutrophil elastase activity relative to the untreated control (indicated by asterisks above individual bars) and to evaluate changes over time within each *C. albicans* strain (indicated by asterisks connecting the corresponding bars). The results were considered statistically significant for p < 0.05 (*p < 0.05, **p < 0.01, ***p < 0.001, ***p < 0.0001), ns, p > 0.05. For clarity, ns differences (p > 0.05) are not marked in the figure. The experiment was repeated five times independently.

The suppression of NET formation and ROS generation, along with increased neutrophil survival in response to mature biofilms, suggests that alternative immune response mechanisms may be activated. Another potential strategy for pathogen elimination is degranulation, which involves the release of proteolytic enzymes, such NE, into the infection site. These mechanisms are less dependent on oxygen availability, making them potentially more effective in the hypoxic microenvironment of mature biofilms. NE activity was measured in supernatants after 3 hours of neutrophil incubation with biofilms at different stages of development (6, 24, and 48 hours). The analysis focused on wild-type strains, as previous results indicated that ATCC 10231 and SC5314 caused the most significant alterations in neutrophil responses, including the strongest inhibition of NETosis and ROS production. The results presented in **Figure 9** indicate that the activity of neutrophil elastase in the supernatant increases in response to *C. albicans* biofilms, with a striking correlation between biofilm maturity and the amount of elastase released. This contrasts with previous results, where we observed inhibition of ROS and NETosis in the presence of denser biofilms, suggesting that while ROS-dependent pathways may be hindered by biofilm structure, other neutrophil responses such as degranulation are instead amplified. Importantly, in the case of mature biofilms, the detected neutrophil elastase is unlikely to originate from NETosis, as NET formation was nearly absent in these conditions. Instead, the increase in elastase activity likely reflects a shift toward direct degranulation as an alternative neutrophil response to biofilm exposure.

### 3.5 Targeting HIF-1α as a potential strategy for modulating neutrophil responses

**Figure 10:**
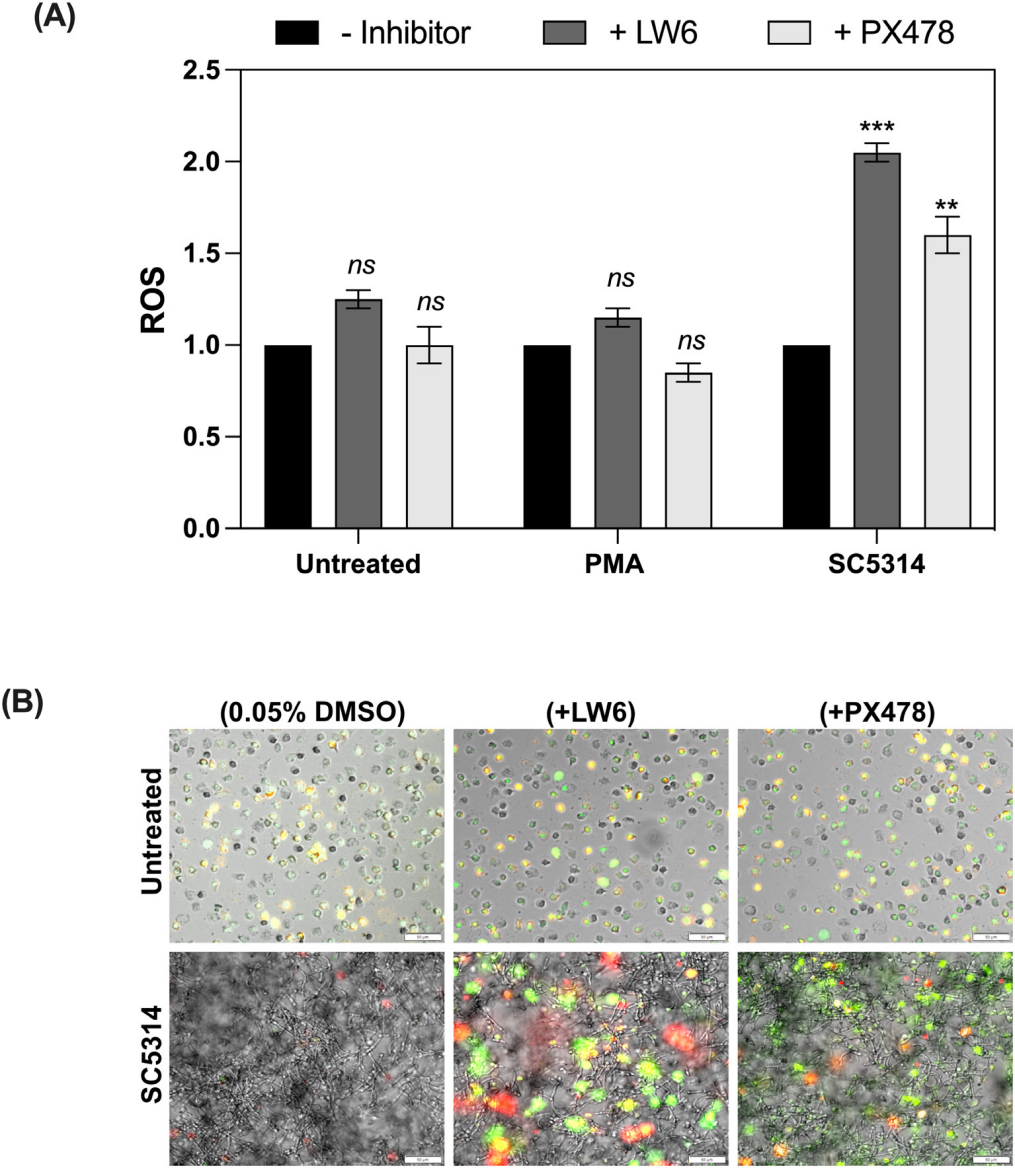
The impact of HIF-1α inhibitors on ROS production and NET formation by neutrophils in the presence of *C albicans* SC5314 biofilms. **(A)** ROS production in neutrophils treated with 2 μM LW6, 10 μM PX478 or after exposure to *C albicans* biofilms for 1 hour, quantified using DHR123 fluorescence measurement. A representative result from five independent biological experiments is shown. The results were considered statistics significant for p < 0.05 (**p < 0.01, ***p < 0.001) and ns for p > 0.05. **(B)** Representative fluorescence microscopy images of NET formation in neutrophils exposed to *C albicans* biofilms with or without HIF-1α inhibitors. NETs were visualized using Sytox Green staining, along with primary anti-citrullinated histone H3 antibodies, followed by detection with Alexa Fluor 555-conjugated secondary antibodies. The images present representative merged channel images. The experiment was repeated five times independently.

HIF-1α, as a master regulator of signaling pathways involved in physiological and pathological processes, has emerged as a promising therapeutic strategy for various diseases (32). To investigate the effects of HIF-1α on neutrophil response to fungal biofilm-associated infections, two inhibitors: LW6 - (Aryloxyacetylamino)benzoic acid analogue and PX478 - (S-2-amino-3-[4′-N,N,-bis (2-chloroethyl) amino]-phenyl propionic acid N-oxide dihydrochloride) were selected. LW6 promotes HIF-1α degradation by enhancing the activity of the von Hippel–Lindau (VHL) protein, which facilitates ubiquitin-mediated proteasomal degradation (33). PX478 reduces HIF-1α levels by inhibiting its synthesis and stabilizing its polyubiquitinated form, leading to enhanced proteasomal degradation (34). In our study, we focused on assessing the effect of HIF-1α inhibition on NETosis and ROS production, because these immune responses are significantly suppressed in the presence of biofilms formed by *C. albicans*. The effect of the selected inhibitors on HIF-1α levels was verified during the optimization stage using Western blot analysis (**Supplementary Figure 6**). In these experiments, neutrophils were pre-incubated with the selected inhibitors to allow intracellular uptake. Subsequently, the pretreated neutrophils were added to mature 48-hour biofilms formed by the *C. albicans* SC5314 strain. This strain was selected due to its higher virulence and our earlier experiments showed that biofilms formed by SC5314 lead to the highest accumulation of HIF-1α. ROS production was analyzed after 1 hour of incubation (**Figure 10A**), while NETosis was assessed after 6 hours (**Figure 10B**). ROS measurement results were normalized independently within each experimental group. For neutrophils without biofilm exposure, the untreated control condition (+0.05% DMSO) was set as the baseline (value = 1), and the effect of inhibitors was evaluated relative to this baseline. For neutrophils exposed to biofilms, the untreated condition (without inhibitors) was set as the baseline (value = 1), and the effects of inhibitors were evaluated relative to this biofilm baseline. Treatment of control neutrophils with LW6 led to a slight increase in ROS production compared to the untreated control group. PX478 did not significantly affect ROS levels in the control group (**Figure 10A**). Notably, the presence of SC5314 biofilms markedly amplified the effect of both inhibitors, leading to a substantial increase in ROS generation. Similarly, NET formation was only slightly elevated in the control group following treatment with LW6 and PX478 but showed a pronounced increase when neutrophils were exposed to SC5314 biofilms, highlighting the impact of these inhibitors on enhancing NETosis under biofilm conditions (**Figure 10B**).

## 4 Discussion

Biofilms are highly structured microbial communities that play a central role in the persistence and chronicity of various infections, including those caused by *C. albicans* (1, 4, 35). Although studies on isolated virulence factors provide valuable insights into the mechanisms of pathogenesis and host-pathogen interactions, recent studies have shown that the immune response to biofilms is more intricately than previously thought, with evidence suggesting that certain immune defense mechanisms may become inactive or suppressed. However, the underlying mechanisms driving these alterations, particularly in neutrophil responses, remain poorly understood.

The analysis of actions of individual factors, such as aspartic proteases, mannans, glucans or toxins allows for precise determination of their role in the immune response under well-controlled conditions. However, in an infectious environment, these factors interact with immune cells within the specific microenvironment of the biofilm, which evolves as the infection progresses. Biofilms are not homogeneous structures—they exhibit strong physicochemical gradients, including variations in nutrient availability, metabolite accumulation, and gas diffusion, all of which can significantly influence immune responses (24, 36). As neutrophils infiltrate deeper layers of the biofilm, they are increasingly exposed to specific conditions, which may shape their functional responses. One of the key factors shaping the biofilm microenvironment is oxygen availability. As biofilms mature, the high density of microbial cells leads to increased metabolic activity, resulting in the rapid consumption of available oxygen (21, 24). As demonstrated in this study, oxygen level may be strongly dependent on the biofilm’s maturity and the specific characteristics of *C. albicans* strains. The significantly reduced oxygen levels observed in mature biofilms suggest that dynamic metabolic changes, along with the presence of the ECM, may limit gas diffusion, ultimately modulating immune cell functions within deeper biofilm layers. The biofilm ECM is essential for maintaining structural stability and regulating oxygen diffusion. In *C. albicans* biofilms, ECM is composed of high-molecular-weight polysaccharides, including a significant portion (87%) of α-1,2 branched and α-1,6 mannans. This mannan interacts with linear β-1,6 glucan (constituting about 13%), forming a mannan–glucan complex, which is distinct from the polysaccharide structures found in the fungal cell wall (37–39). The role of ECM in forming localized hypoxia is supported by our findings from biofilms formed by the *Δmnn9* strain, which exhibits impaired mannan synthesis due to the lack of a dense mannan network, a major component of the ECM in *C. albicans* biofilms (39), correlates with higher oxygen levels within the biofilm compared to wild-type strains. Notably, the oxygen levels in *Δmnn9* biofilms were comparable to those observed in the *Δefg1/Δcph1* strain, which does not form biofilms and remains in a planktonic state. XTT metabolic assays did not reveal drastic differences in metabolic activity between the tested strains, suggesting that the oxygen levels in *Δmnn9* biofilms are more likely influenced by increased permeability rather than reduced fungal metabolism. In the case of the second tested mutant, *Δbgl2*, which is defective in β-glucan synthesis, small differences in oxygen levels were observed compared to wild-type strains, but they were less pronounced than in the *Δmnn9* mutant. This likely reflects the lower abundance of glucans within the ECM, making their contribution to gas diffusion limitations less significant compared to mannans.

Using an *in vitro* model, we demonstrated that human neutrophils in contact with fully developed *C. albicans* biofilms experience a significant increase in intracellular hypoxia. Notably, the SC5314 strain, known for its higher virulence, induced a greater hypoxic response in neutrophils compared to the ATCC 10231 strain. The SC5314 strain generally forms more dense biofilms and has higher metabolic activity, which may explain its increased oxygen consumption (40). These findings align with our results indicating that the availability of oxygen in biofilms depends not only on their maturation stage but also on strain-specific characteristics. Neutrophils exposed to *Δbgl2* mutant biofilms exhibited lower hypoxia levels compared to wild-type biofilms, indicating that defects in glucan synthesis reduce, but do not abolish, the biofilm’s ability to induce hypoxia. In contrast, *Δmnn9* biofilms triggered significantly lower hypoxia levels at all developmental stages, confirming the role of ECM in shaping the biofilm microenvironment. Similarly, the *Δefg1/Δcph1* strain, which does not form biofilms and remains in a planktonic state, failed to induce hypoxia in neutrophils, further emphasizing the crucial role of biofilm architecture in oxygen depletion and the subsequent modulation of neutrophil responses.

Furthermore, we demonstrated that biofilm-induced hypoxia contributed to the stabilization of HIF-1α in neutrophils. The significant increase in HIF-1α levels in response to wild-type strains suggests that biofilm may alter neutrophil functionality through HIF-1α-dependent signaling pathways (41). Previous research has demonstrated that HIF-1α can exert both stimulatory and suppressive effects on immune cells, with its influence varying depending on the cell type involved (25, 42, 43).

One of the key characteristics of neutrophils is their intrinsic ability to undergo constitutive apoptosis, a mechanism essential for maintaining tissue homeostasis (44). Our findings indicate that exposure to *C. albicans* biofilms significantly enhances neutrophil survival compared to unstimulated cells, which correlates with a marked suppression of caspase-3/7 activation. This effect was also observed in biofilms formed by the *Δbgl2* mutant, while the inhibition was less pronounced in the case of the *Δmnn9* strain. One potential mechanism contributing to the prolonged neutrophil lifespan within the biofilm is the sustained expression of HIF-1α, in line with previous reports suggesting that HIF-1α suppresses apoptosis through NF-κB pathway activation (25, 45). Furthermore, the enhanced neutrophil survival correlated with increased accumulation of Mcl-1, a key anti-apoptotic protein crucial for neutrophil viability during infection (46). Studies on HepG2 cells have shown that the *MCL1* gene contains a hypoxia-responsive element (HRE) capable of binding HIF-1α (47), suggesting that its expression may be regulated by HIF-1α under hypoxic conditions. The link between HIF-1α and neutrophil apoptosis inhibition under low oxygen levels has also been demonstrated in a mouse model (25). Our findings also demonstrate a significant increase in MIP-1β production by neutrophils in response to *C. albicans* wild-type biofilms, particularly after prolonged mutual contact. This observation aligns with the well-known role of MIP-1β as a key chemokine involved in immune cell survival and recruitment, especially under stress conditions such as hypoxia (25). Additionally, we observed an increase in IL-8 levels, further supporting the idea that biofilm-exposed neutrophils enhance the secretion of chemokines involved in immune cell recruitment and inflammation.

While hypoxia is the primary driver of HIF-1α stabilization, alternative mechanisms, such as microbial ion chelation, may also play a role by modulating HIF-1α degradation independently of oxygen levels (48). Iron chelators are known to stabilize HIF-1α by inhibiting prolyl hydroxylases, which require iron as a cofactor for hydroxylation and subsequent degradation of HIF-1α. However, in our study, the observed increase in neutrophil HIF-1α expression correlated strongly with oxygen consumption within biofilms, indicating that hypoxia remains probably the dominant factor (48).

We subsequently focused on the impact of biofilm-induced hypoxia on the immunological mechanisms employed by neutrophils to eliminate fungal cells. In particular, we focused on NETosis, a key neutrophil defense strategy. The ability to activate this mechanism under conditions of low oxygen concentration is not entirely clear. For example, in the work of Branitzki-Heinemann et al., hypoxic conditions were shown to completely disable the response to PMA and heat-inactivated *Staphylococcus aureus* but did not significantly affect the response to live bacteria (49). Our findings reveal that NETosis is prominently active when neutrophils encounter biofilms at an early stage of development. However, as the biofilms mature and oxygen levels decrease, we observed a progressive silencing of this mechanism. This correlation was disturbed in the case of the *Δmmn9* mutant, which activated the release of NETs regardless of the stage of biofilm development and incubation time. The results align with the findings of Johnson et al., where it was demonstrated that a *C. albicans* mutant (*pmr1Δ/Δ*), which is defective in mannan production, also activated increased NETosis (19). Our findings suggest a broader role for mannan in NETosis inhibition, not only as components that mask cell wall epitopes as suggested by Johnson et al., but also through its effects on oxygen penetration and hypoxia generation. The observed in our experiment reduction in NETosis was closely associated with the diminished production of ROS in contact with biofilms. ROS serve as a crucial trigger initiating NET formation (11). Literature reports suggest that ROS generation under hypoxia is probably impaired due to the lack of available molecular oxygen (49, 50). Therefore, the progressive hypoxia induced by maturing biofilms likely compromises ROS production, thereby inhibiting NETosis in neutrophils. The observed suppression of NETosis and ROS production in our study, when neutrophils interact with *C. albicans* biofilms, might not represent a detrimental effect imposed by the biofilm but could instead reflect a physiological adaptation to these low-oxygen conditions. In hypoxic environments, neutrophils may adapt by prioritizing defense strategies that are less dependent on oxygen, potentially shifting away from ROS-dependent mechanisms. One such alternative response is degranulation. Interestingly, ROS have been shown to inhibit signal mediators of degranulation, suggesting a reciprocal regulation between these two pathways (51). In our experiments, we observed high level of neutrophil elastase activity in supernatants collected after contact of neutrophils with mature *C. albicans* biofilms. Notably, NETosis was not observed under these conditions, confirming that the detected elastase was released during degranulation. The results are consistent with previous reports in the literature, which also demonstrate that degranulation may be a key defense mechanism of neutrophils under hypoxic conditions (52). The combination of prolonged neutrophil survival suppressed ROS and NETosis, and enhanced degranulation may have profound consequences for host-pathogen interactions. While neutrophil elastase is crucial for pathogen elimination, it also degrades extracellular matrix components and host tissues, contributing to chronic inflammation and tissue damage (52). Thus, in the hypoxic biofilm environment, prolonged neutrophil viability and increased degranulation may exacerbate inflammation and tissue injury, particularly due to the sustained release of elastase and pro-inflammatory cytokines (52). Moreover, studies have demonstrated that *C. albicans* can exploit neutrophil elastase to enhance invasion, as the enzyme interacts with fungal adhesins and facilitates epithelial penetration (53). This suggests that in the biofilm microenvironment, where elastase release is significantly elevated, the balance between fungal clearance and potential pathogen-driven invasion is particularly delicate.

Inhibitors of HIF-1α have received significant attention in recent years due to their potential to modulate the hypoxic response in diseases such as cancer, where hypoxia is a common feature of the microenvironment and contributes to tumor growth, metastasis, and resistance to therapy (32). By blocking HIF-1α activity, inhibitors can disrupt the hypoxia-driven survival mechanisms in tumor cells, potentially enhancing the effectiveness of conventional treatments. Given the essential role of HIF-1α in neutrophil survival and adaptation to hypoxia, we investigated whether pharmacological inhibition of this factor could alter some of the biofilm-induced immunological responses. Our results with the HIF-1α inhibitors LW6 and PX478 suggest a potential role in modulating neutrophil responses within the biofilm microenvironment, although their effects were not uniform and require further validation. LW6 appeared to enhance ROS production, which in turn was associated with a partial restoration of NETosis activity. A similar but less pronounced trend was observed with PX478, indicating that HIF-1α modulation may play a role in neutrophil response but with varying degrees of effectiveness. These findings align with studies on the A549 cell model, where LW6 was shown to enhance superoxide production (54). However, while targeting HIF-1α may be beneficial in counteracting some biofilm-induced immune alterations, it is essential to balance potential improvements in neutrophil function with the risk of excessive ROS production and inflammation. HIF-1α is essential for cellular adaptation to hypoxia, supporting metabolic processes and cell survival, and its complete suppression may disrupt these beneficial adaptations. Therefore, further research is necessary to optimize therapeutic strategies that selectively modulate HIF-1α activity to restore immune function without exacerbating inflammation or tissue damage.

## Data Availability

The datasets presented in this study are available in the RODBUK Cracow Open Research Data repository under the following DOI: https://doi.org/10.57903/UJ/7GQAP4.
