# Supplementary material for: The hypoxic microenvironment of Candida albicans biofilms shapes neutrophil responses
Source: Front Immunol. 2025 Apr 22;16:1547559. doi: 10.3389/fimmu.2025.1547559 (PMC12053278; doi:10.3389/fimmu.2025.1547559)
Supplement: Supplementary file 1 [file DataSheet1.pdf]

Supplementary Material

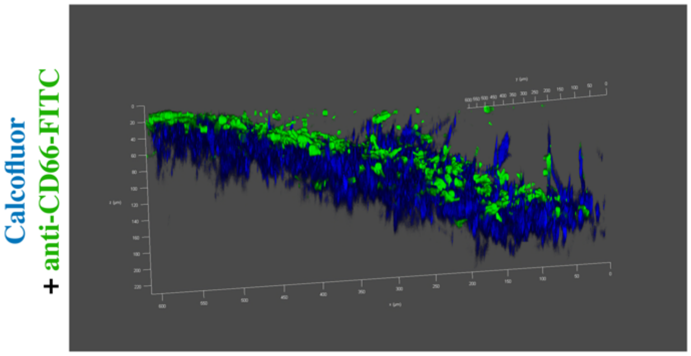

Supplementary Figure 1. Analysis of neutrophil interaction with *C. albicans* biofilm. The presented images show neutrophil interaction with a 48-hour *C. albicans* ATCC 10231 biofilm. Neutrophils were labeled using anti-CD66abce-FITC antibodies (1:1000), while the C. albicans cell wall was stained with Calcofluor (DAPI channel). Microscopy images were acquired using a Stellaris 5 confocal microscope (Leica), and projections along the X, Y, and Z axes were generated using Leica LAS X software.

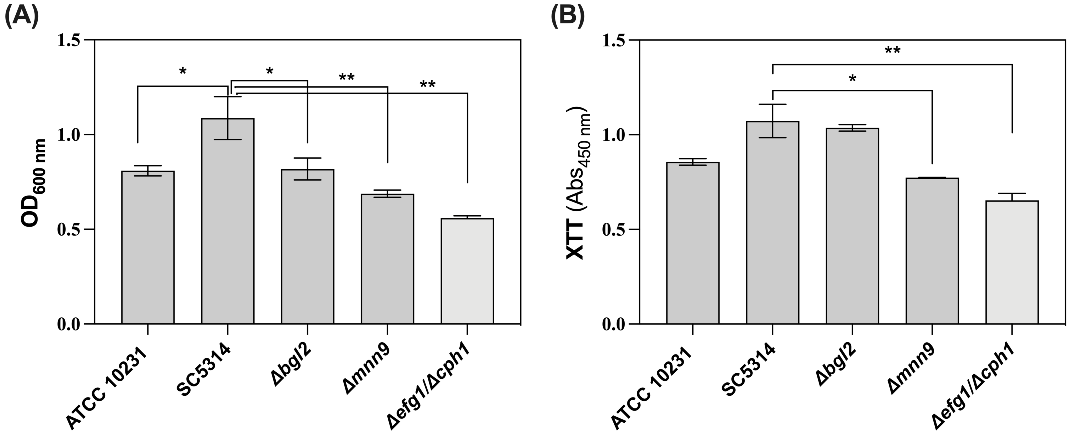

Supplementary Figure 2. Comparison of optical density and metabolic activity of biofilms formed by different *C. albicans* strains, including ATCC 10231, SC5314, *Δbgl2*, and *Δmnn9*, *Δefg1/Δcph1*. Biofilms (initial cell density of 10^5/ml, 100 µl RPMI) were grown for 48 hours in 96-well plates under standard conditions. After incubation, OD_{600} was measured to assess biofilm biomass (A), and the XTT assay was performed to analyze the metabolic activity of biofilms (B). Statistical significance was evaluated using multiple comparison tests, analyzing differences among all groups. The results were considered statistically significant for $p < 0.05$ (* $p < 0.05$, ** $p < 0.01$). For clarity, ns differences ($p > 0.05$) are not marked in the figure.

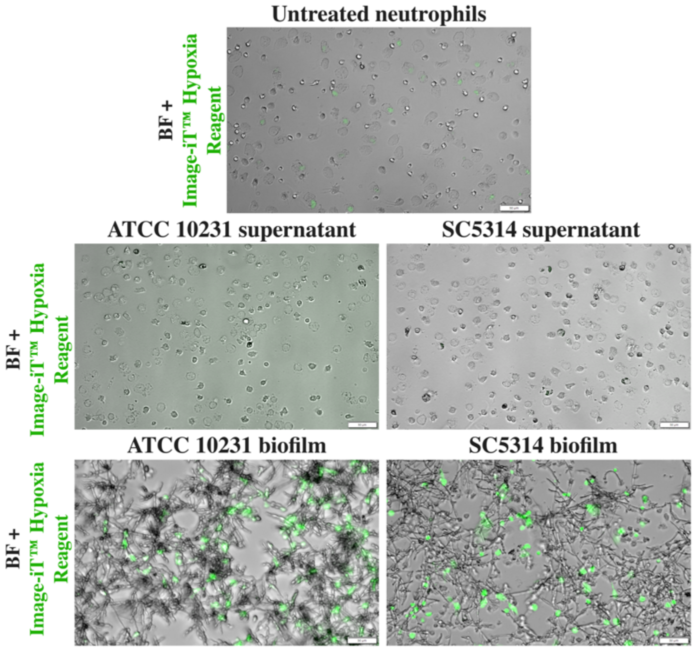

Supplementary Figure 3. **Assessment of hypoxia induction in neutrophils exposed to *C. albicans* biofilms and biofilm-derived supernatants.** Neutrophils were pre-labeled with the Image-iT™ Hypoxia Reagent and then incubated either with the cell-free supernatant collected from 48-hour biofilms of *C. albicans* ATCC 10231 and SC5314 or directly with the corresponding biofilms. The incubation was carried out for 2 hours, after which images were acquired at 60× magnification using an Olympus IX73 microscope. Brightfield (BF) and hypoxia reagent fluorescence are shown. Green fluorescence indicates hypoxic neutrophils. No hypoxia signal was observed when neutrophils were incubated with biofilm-derived supernatants, while a pronounced hypoxic response was detected in neutrophils interacting with mature biofilms.

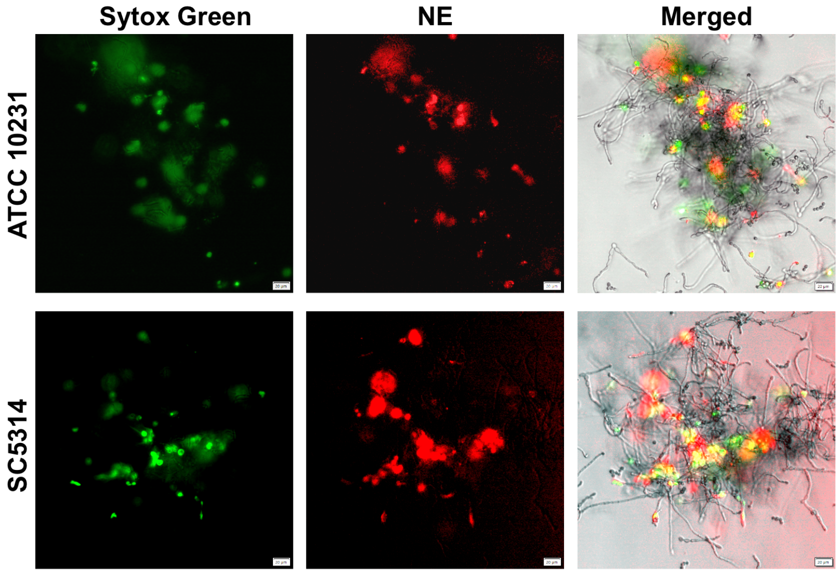

Supplementary Figure 4. Analysis of colocalization of neutrophil elastase (NE) with DNA in NETs after neutrophil exposure to *C. albicans* biofilms. DNA was stained with Sytox Green (green), while neutrophil elastase (NE) was visualized using specific primary antibodies anti-neutrophil elastase and anti-rabbit secondary antibodies (Alexa Fluor 555). The merged images reveal colocalization of NE with extracellular DNA structures in biofilm conditions, confirming the formation of neutrophil extracellular traps (NETs).

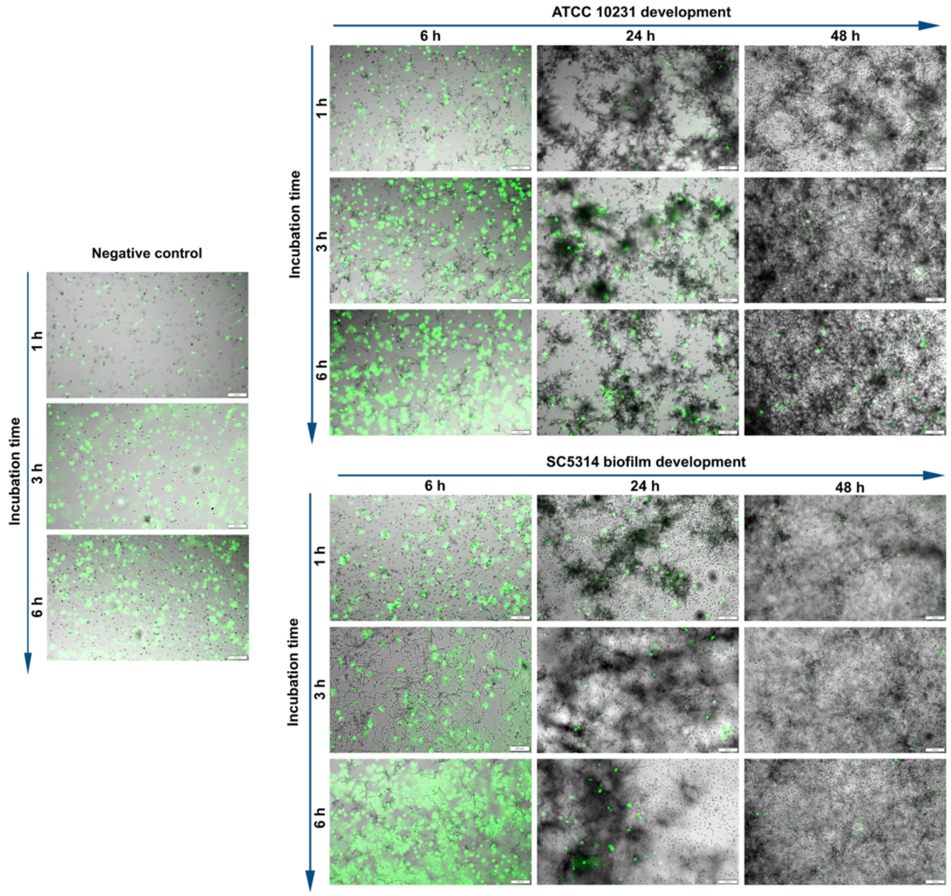

Supplementary Figure 5. Analysis of NETs formation in response to *C. albicans* biofilm – images without washing steps and antibody staining. Neutrophils were incubated with *C. albicans* biofilms formed by ATCC 10231 and SC5314 strains at different stages of biofilm development (6, 24, and 48 hours). NETs formation was assessed using fluorescence microscopy with Sytox Green nucleic acid dye (green channel). Bright-field (BF) microscopy was used to visualize biofilm structure.

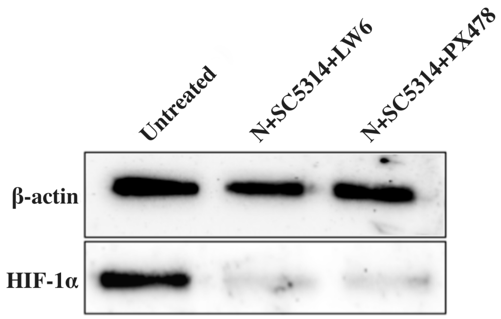

Supplementary Figure 6. Analysis of the impact of 2 µM LW6 and 10 µM PX478 on HIF-1α protein levels, assessed using Western Blot (representative result).
